# Inhibition of astrocyte hemichannel improves recovery from spinal cord injury

**DOI:** 10.1172/jci.insight.134611

**Published:** 2021-03-08

**Authors:** Chao Zhang, Zhao Yan, Asif Maknojia, Manuel A. Riquelme, Sumin Gu, Grant Booher, David J. Wallace, Viktor Bartanusz, Akshay Goswami, Wei Xiong, Ningyan Zhang, Michael J. Mader, Zhiqiang An, Naomi L. Sayre, Jean X. Jiang

**Affiliations:** 1 Department of Biochemistry and Structural Biology, University of Texas Health Science Center at San Antonio, San Antonio, Texas, USA.; 2 The Second Xiangya Hospital of Central South University, Changsha, China.; 3 Department of Neurosurgery, University of Texas Health Science Center at San Antonio, San Antonio, Texas, USA.; 4 Texas Therapeutics Institute, Brown Foundation Institute of Molecular Medicine, University of Texas Health Science Center at Houston, Houston, Texas, USA.; 5 Audie L. Murphy VA Hospital, South Texas Veteran’s Health Care System, San Antonio, Texas, USA.

**Keywords:** Therapeutics, Neurological disorders

## Abstract

Spinal cord injury (SCI) causes severe disability, and the current inability to restore function to the damaged spinal cord leads to lasting detrimental consequences to patients. One strategy to reduce SCI morbidity involves limiting the spread of secondary damage after injury. Previous studies have shown that connexin 43 (Cx43), a gap junction protein richly expressed in spinal cord astrocytes, is a potential mediator of secondary damage. Here, we developed a specific inhibitory antibody, mouse-human chimeric MHC1 antibody (MHC1), that inhibited Cx43 hemichannels, but not gap junctions, and reduced secondary damage in 2 incomplete SCI mouse models. MHC1 inhibited the activation of Cx43 hemichannels in both primary spinal astrocytes and astrocytes in situ. In both SCI mouse models, administration of MHC1 after SCI significantly improved hind limb locomotion function. Remarkably, a single administration of MHC1 30 minutes after injury improved the recovery up to 8 weeks post-SCI. Moreover, MHC1 treatment decreased gliosis and lesion sizes, increased white and gray matter sparing, and improved neuronal survival. Together, these results suggest that inhibition of Cx43 hemichannel function after traumatic SCI reduces secondary damage, limits perilesional gliosis, and improves functional recovery. By targeting hemichannels specifically with an antibody, this study provides a potentially new, innovative therapeutic approach in treating SCI.

## Graphical abstract

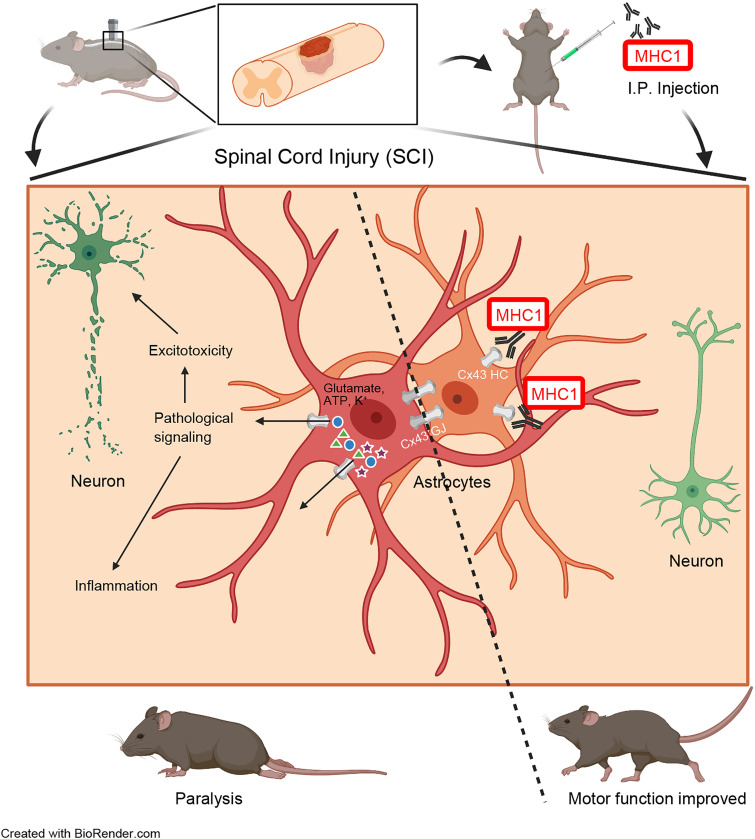

## Introduction

Spinal cord injury (SCI) results in approximately 13,000 admissions to hospitals in the United States and between 250,000 and 500,000 injuries worldwide each year (1). After the initial primary injury, secondary damage occurs, when edema, loss of blood flow, metabolic crisis, and spreading depolarizations cause the area of spinal cord damage to increase (2). These processes elicit increased levels of extracellular molecules and ions, which include glutamate, lactate, K^+^, nitric oxide, arachidonate, reactive oxygen species, and ammonia. The increased extracellular levels of these species in turn create a cytotoxic postinjury environment (3, 4). These factors are known to contribute to increased lesion size after SCI (5, 6). The inability of axons to regrow across the injured site is also a significant complication that hampers recovery. However, most SCI patients have incomplete SCI, in which a portion of spinal cord function is retained (7, 8). While strategies that promote axonal growth are limited, other treatment paradigms that limit secondary damage in incomplete SCIs can potentially allow patients to retain greater function postinjury. Additionally, secondary injury results in glial scar formation and neuronal cell death (3). These can be detrimental to axon regeneration and contribute to other complications that arise from SCI, such as neuropathic pain and spasticity (9). Because strategies that regenerate axons are limited, therapeutically preventing secondary damage after SCI represents a significant strategy in improving patient outcomes.

Connexin 43 (Cx43) protein forms gap junctions that connect adjacent cells and hemichannels that open to the extracellular space; it is primarily located on the surface of astrocytes in the spinal cord (4, 10). Previous studies have shown that Cx43 expression is upregulated after SCI in rodents (4, 11, 12). Huang and colleagues reported that in an astrocyte Cx43-deficient mouse model, the acute inflammatory response and traumatic lesions were reduced (13). Moreover, suppression of Cx43 expression by antisense oligodeoxynucleotide reduced inflammation and improved functional recovery after SCI (14). These studies investigated a role for Cx43 in SCI but did not differentiate between Cx43 in gap junctions and Cx43 in hemichannels. Therefore, the specific role of Cx43 hemichannels versus Cx43 gap junctions in contributing to damage after SCI remains elusive.

Cx43 hemichannel opening is expected to increase secondary injury by releasing components that contribute to the postinjury cytotoxic milieu. Therefore, we investigated the role of Cx43 hemichannels in SCI. We developed a potentially novel monoclonal antibody, MHC1, that targets Cx43 and specifically inhibits hemichannel opening. Importantly, this antibody has no effect on gap junction function. We compared the efficiencies of this antibody in 2 partial SCI mouse models. Results showed that single administration of this antibody within 30 minutes after SCI significantly decreased secondary injury, improved locomotion function, attenuated gliosis, preserved white and gray matter, and protected neurons. These results suggest that antibodies that prevent opening of Cx43 hemichannels represent a new therapeutic strategy for treating SCI.

## Results

### MHC1 antibody specifically inhibits the opening of Cx43 hemichannels.

**Figure 1:**
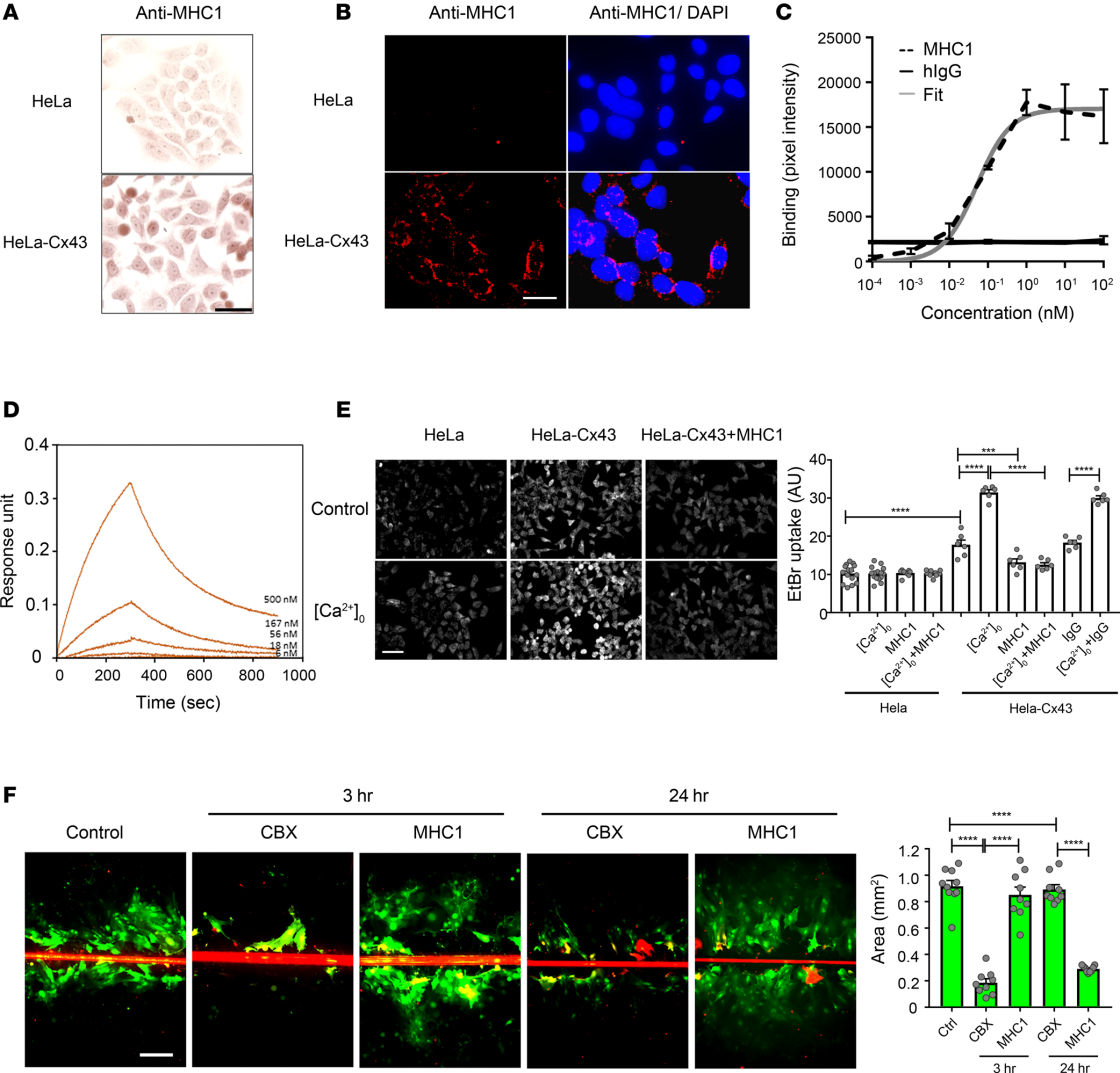
MHC1 antibody binds Cx43 and inhibits the opening of Cx43 hemichannels. (**A** and **B**) Fixed parental HeLa cells or HeLa cells stably transfected with Cx43 were incubated with MHC1 antibody and then labeled with HRP-conjugated anti-human IgG (**A**) or rhodamine-conjugated anti-human IgG and counterstained with DAPI (**B**). Scale bars: 50 μm (**A**), 30 μm (**B**). (**C**) The binding affinity of MHC1 and IgG control to Cx43 peptide was determined by ELISA. *n* = 4. (**D**) Kinetics of MHC1 binding to the Cx43 extracellular domain peptide (N′-CFLSRPTEKTI) as assessed using an Octet RED96. (**E**) Parental HeLa cells or HeLa cells stably transfected with Cx43 were incubated with EGTA to remove extracellular Ca^2+^ ([Ca2+ ]_0_) in the absence or presence of MHC1 (66.7 nM) or control IgG (66.7 nM) before dye uptake assay with 15-minute treatment of 50 μM ethidium bromide (EtBr). (**F**) Primary astrocytes isolated from rat cortical brain were incubated for 3 or 24 hours with MHC1 (66.7 nM) or CBX (100 μM) before scrape-loading dye transfer assay was performed with Lucifer yellow (1%) and rhodamine dextran (1%) for 5 minutes. The level of EtBr dye uptake in **E** and dye transfer in **F** was determined by fluorescence microcopy and quantified by NIH ImageJ software. Scale bar: 200 μm (**E** and **F**). Data are presented as mean ± SEM of 3 independent experiments; each experiment had 2–3 repeats (2–3 wells). Michaelis-Menten equation was used in statistical analysis model (**C**). General linear model was used in statistical analysis (**E** and **F**). ***P < 0.001; ****P < 0.0001.

MHC1 is a mouse-human chimeric antibody against Cx43, which contains the mouse variable domains and human constant domains (see Methods). We tested the specificity of MHC1 to Cx43 using HeLa-Cx43 cells, which are stably transfected with Cx43. Immunocytochemistry with horseradish peroxidase (HRP) staining (Figure 1A) and immunofluorescence (Figure 1B) showed that HeLa-43 cells, in contrast to parental HeLa cells that do not express Cx43, were positively labeled with MHC1 antibody. Cx43 was detected along the cell border of HeLa-Cx43 cells (Figure 1B). MHC1 antibody bound to Cx43 present on the cell surface of attached or suspended MLO-Y4 cells (Supplemental Figure 1; supplemental material available online with this article; https://doi.org/10.1172/jci.insight.134611DS1). Next, we measured the specific binding of MHC1 to the Cx43 extracellular domain peptide by using ELISA (Figure 1C) and Bio-layer interferometry (Octet RED96) (Figure 1D) approaches. ELISA measured the binding affinity at equilibrium, with maximal binding at 15,018 ± 663.5, and the EC_50_ of MHC1 binding to the peptide was 7.63 × 10^–2^ ± 2.19 × 10^–2^ nM (Figure 1C). Bio-layer interferometry via Octet measures the dynamic binding affinity with both an on and off rate. The *K_D_* of MHC1 binding to the peptide as measured by Octet was 421 ± 7 nM (Figure 1D).

Cx43 hemichannels are induced to open in response to low extracellular Ca^2+^ and Mg^2+^ (15). We incubated the parental HeLa and HeLa-Cx43 cells with normal culture medium or Ca^2+^ and Mg^2+^-free medium and measured Cx43 hemichannel activity by testing uptake of EtBr into cells (16). In parental HeLa cells, low and normal Ca^2+^/Mg^2+^ and MHC1 had no effect on EtBr signals. In contrast, hemichannels in HeLa cells stably expressing Cx43 were open after depletion of extracellular Ca^2+^/Mg^2+^, as measured by increased EtBr signal. This opening was significantly inhibited by a 30-minute preincubation with MHC1 antibody (66.7 nM). In contrast, IgG (66.7 nM) preincubation had no effect on hemichannel opening (Figure 1E). Furthermore, the activity of hemichannels in medium containing normal levels of Ca^2+^ and Mg^2+^ (1.8 mM) was also significantly inhibited by MHC1 antibody. To test whether MHC1 had an effect on the function of Cx43-containing gap junctions, the scrape-loading dye transfer assay was performed in rat cortical astrocyte primary cultures. In brief, cells were incubated with a gap junction–permeant dye, and the dye was loaded into a single line of astrocytes via scraping with a razor blade for 5 minutes. In scrape-loaded astrocytes, the dye traveled through functional gap junctions into adjacent gap junction–coupled astrocytes that avoided the scrape-loading. Astrocytes were pretreated with MHC1 (66.7 nM) or a known gap junction blocker, carbenoxolone (CBX) (100 μM), for either 3 or 24 hours at 37°C prior to the gap junction coupling assay. The results showed that treatment of MHC1, even for an extended 24-hour treatment, had no effect on gap junction coupling in astrocytes, while the positive control CBX significantly inhibited gap junction coupling (Figure 1F).

We further validated the specificity of MHC1 on Cx43 hemichannels by comparing to pannexin channels, which like connexins, form hemichannels on the cell surface. We used MDA-MB231 cells that express pannexin 1 but minimal Cx43 (17, 18). Pannexin 1 channels are responsive to ATP. ATP induced the opening of pannexin 1 channels and uptake of EtBr. This opening was inhibited by CBX (which inhibits both connexin and pannexin channels) and probenecid (which only inhibits pannexin channels) (19, 20); however, MHC1 had no effect on pannexin channels (Supplemental Figure 2).

Inflammatory factors such as IL-1β are reported to activate Cx43 hemichannels (21). IL-1β is also released after SCI (22). To test the effect under conditions more closely relevant to SCI, we tested Cx43 activity after IL-1β treatment in primary astrocytes prepared from mouse spinal cords, similar to our previously published protocols (23). We validated the purity of primary cultured astrocytes using immunocytochemistry for astrocyte markers on early passage astrocytes isolated from the spinal cord and found at least 96% expression of the astrocyte marker glial fibrillary acidic protein (GFAP) and low abundance of nonastrocytic markers (Supplemental Figure 3).

**Figure 2:**
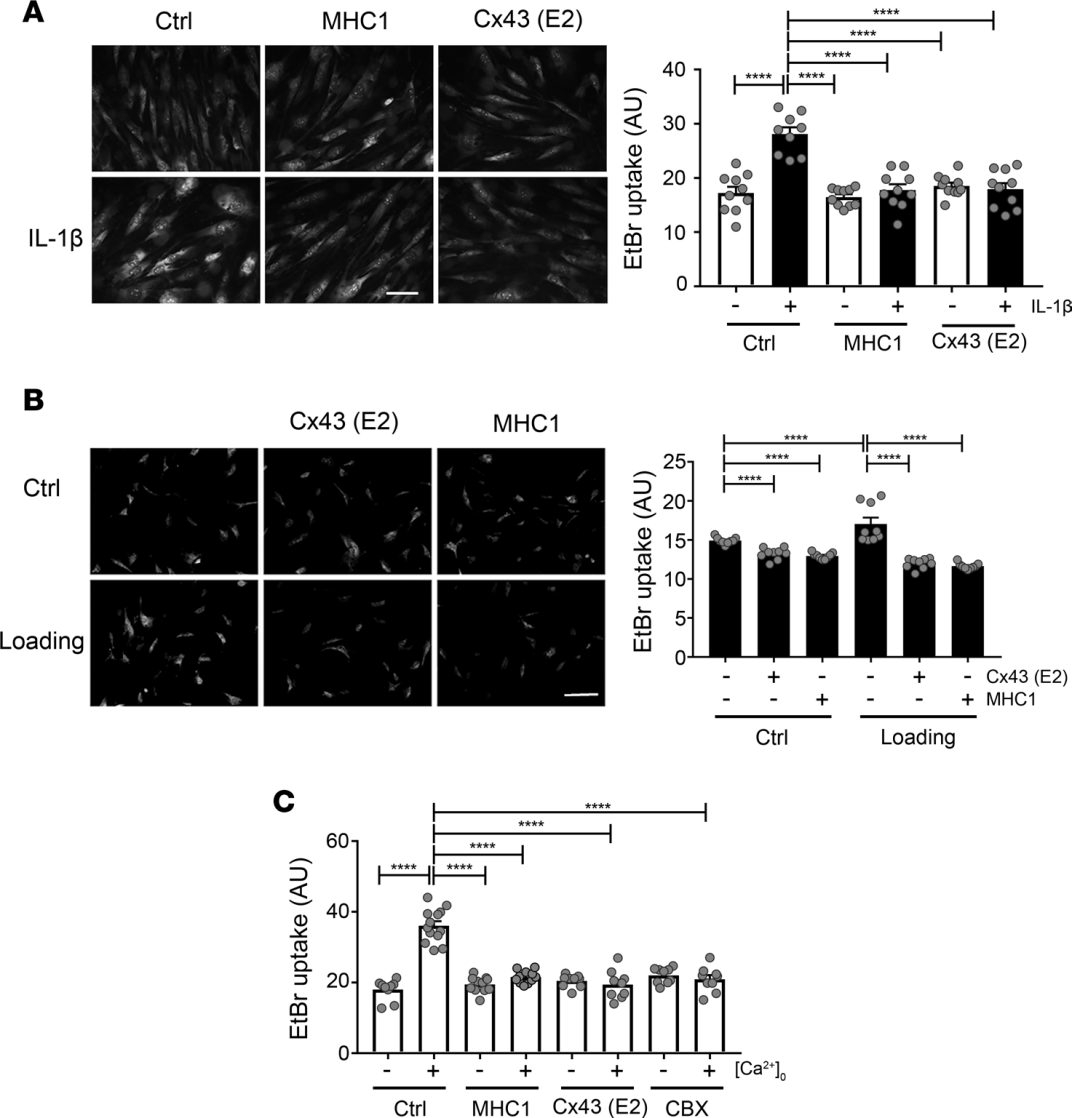
MHC1 antibody inhibits hemichannel opening in astrocytes by cytokine, mechanical stimulation, and low extracellular Ca^2+^/Mg^2+^. (**A**) Primary astrocytes isolated from mouse spinal cord were treated with IL-1β (1 nM) for 24 hours. MHC1 (66.7 nM) or Cx43 (E2) antibody (6.7 nM) was then added 30 minutes prior to dye uptake assay with EtBr (50 μM) for 5 minutes. (**B**) Primary astrocytes isolated from mouse spinal cord were subjected to mechanical loading by Flexcell device in the absence or presence of MHC1 (66.7 nM) or Cx43E2 antibody (20 nM), and after loading, dye uptake assay was performed with EtBr (50 μM) for 5 minutes. (**C**) Primary astrocytes isolated from mouse spinal cord were incubated with or without EGTA to remove or maintain extracellular Ca^2+^/Mg^2+^ ([Ca^2+^ ]_0_) in the absence or presence of MHC1 (66.7 nM), Cx43E2 antibody (6.7 nM) or CBX (100 μM) for 30 minutes before dye uptake assay with EtBr (50 μM) for 15 minutes. Plus sign represents the presence of EGTA and absence of [Ca^2+^ ]_0_. Minus sign represents the absence of EGTA and presence of [Ca^2+^ ]_0_. The level of EtBr dye uptake was determined by fluorescence microcopy and quantified by NIH ImageJ software. The results are presented as mean ± SEM of 3 independent experiment, each experiment had 3–5 repeats (3–5 wells). Scale bar: 100 μm (**A** and **B**). General linear model was used in statistical analysis (**A**–**C**). **P < 0.01, ****P < 0.0001.

Primary spinal cord astrocytes were prepared, and the authenticity of spinal cord astrocytes was verified by the immunolabeling of vimentin and GFAP (Supplemental Figure 3A) and quantification of percentage of cells from spinal cord astrocyte preparation immunolabeled with astrocyte and neuronal markers (Supplemental Figure 3B). Spinal cord astrocyte hemichannel activity, as measured by EtBr uptake, was increased after 24 hours of IL-1 (1 nM) administration (Figure 2A). Preincubation for 15 minutes with Cx43E2 antibody (6.7 nM), a polyclonal hemichannel-blocking antibody developed in-house that specifically binds to second extracellular domain (E2) of Cx43 (24), significantly inhibited hemichannel opening by IL-1β. As with the Cx43E2 antibody, primary spinal cord astrocytes preincubated with MHC1 (66.7 nM) did not increase EtBr uptake in response to IL-1 (Figure 2A). These results suggest that MHC1 functions similarly to Cx43E2 to inhibit Cx43 in astrocytes. We have previously shown that mechanical stress activates Cx43 hemichannels (24, 25). To mimic SCI as a result of mechanical insult in vitro, we cultured the mouse primary spinal cord astrocytes in 6-well Flexcell plates that have elastic bottoms and subjected astrocytes to mechanical loading after preincubation with either MHC1 or Cx43E2 antibody. Mechanical loading introduced by the Flexcell unit opened Cx43 hemichannels, and this opening was inhibited by preincubation with MHC1 or Cx43E2 antibody (Figure 2B). To further validate the specific function of MHC1, mouse spinal cord astrocytes were subjected to low extracellular Ca^2+^/Mg^2+^ to induce the opening of hemichannels. Hemichannel opening was significantly inhibited by MHC1 at comparable levels to CBX or the Cx43E2 antibody (Figure 2C).

### Delivery of MHC1 to spinal cord inhibits opening of hemichannels after acute SCI.

**Figure 3:**
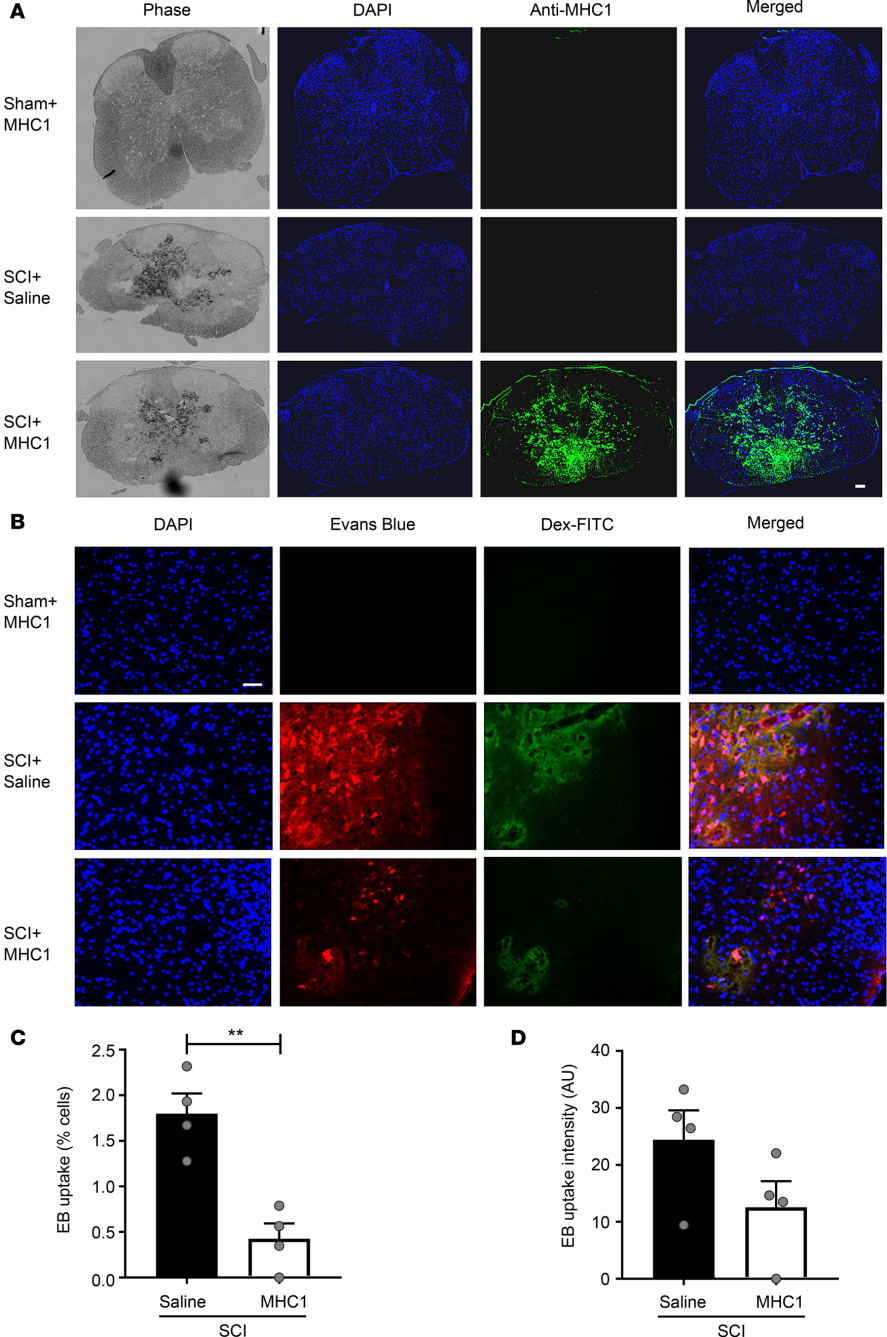
MHC1 delivered to spinal cord inhibits opening of hemichannels after acute SCI. (**A**) MHC1 antibody (25 mg/kg) was i.p. injected 30 minutes after SCI under model 2. Four hours after the injection, spinal cords were isolated and fixed, and frozen tissue sections were prepared and immunolabeled with FITC-conjugated anti-human IgG secondary antibody. Images were taken from injury site. Scale bar: 100 μm. (**B**) MHC1 antibody (25 mg/kg) was i.p. injected 30 minutes after SCI under model 1. Evans blue (EB) and FITC-dextran dye were coinjected through tail vein 4 hours after i.p. injection. Mice were euthanized and perfused before isolation of spinal cords. Frozen tissue sections were prepared and EB dye uptake (red) was detected by fluorescence microscopy. Images were taken from the perilesional area (area < 1.5 mm from injury border). Scale bar: 50 μm. The percentage (**C**) and signal intensity (**D**) of EB-positive cells were quantified by NIH ImageJ software, and the results were combined from injury site, perilesional area, and distal area. The results are presented as mean ± SEM. SCI+Saline (*n* = 4), SCI+MHC1 (*n* = 4). Unpaired *t* test (1 tailed) was used in statistical analysis (**C** and **D**). **P < 0.01.

The blood-spinal cord barrier (BSCB) is a highly selective semipermeable border that separates the circulating blood from the brain and extracellular fluid. This provides a unique microenvironment for the spinal cord (26). The BSCB restricts the diffusion of solutes in the blood (e.g., large or hydrophilic molecules) into the cerebrospinal fluid (27). However, this barrier is disrupted immediately after SCI, becoming permeable to large molecules. To determine whether the MHC1 antibody enters the injury lesion after SCI, we injected MHC1 or saline 30 minutes post-SCI in mice (using SCI model 2, which has a focused impact on spinal cord; see details in Methods). Four hours later, we harvested the spinal cord and measured the anti-human immunoreactivity in the mouse SCI tissue in the site of injury (as shown in images in Figure 3A). Immunolabeling of cryo-sections with anti-human IgG antibody showed positive signal in MHC1-injected samples. However, no positive immunolabeling was observed in samples from mice given SCI plus saline injection or in samples from sham-operated MHC1-treated animals, indicating the MHC1 was able to reach the site of injury (Figure 3A).

We tested whether hemichannels were activated after SCI using an Evans blue dye uptake assay (using SCI model 1, which has a broad impact on spinal cord, see details in Methods). Evans blue has been used to detect hemichannel activity in situ (28). FITC-dextran (MW 10,000), which is too large to pass through hemichannels, was coinjected to exclude nonspecific dye uptake due to cell membrane breakage. We quantified Evans blue signal in 3 discrete regions: within the lesion (SCI, Supplemental Figure 4), in tissue less than 1.5 mm from the injury border (perilesion, Supplemental Figure 5), and in tissue more than 1.5 mm distal to the injury site (Supplemental Figure 6). The total numbers of cells were also quantified via DAPI staining. Uptake of Evans blue dye was measured in FITC-dextran–negative cells. Increased Evans blue uptake was observed in both SCI and perilesional SCI regions. This uptake was significantly inhibited by MHC1 treatment (Supplemental Figures 4 and 5). However, there was minimal Evans blue uptake in the distal SCI region (Supplemental Figure 6). The pattern of hemichannel activity is well correlated with a previous report showing the strongest Cx43 expression near the lesion, especially in the rostral perilesional site, which then gradually decreases with distance (11). Figure 3, C and D, show the combined quantification results from lesion, proximal, and distal regions. The presenting images of dye uptake of these 3 regions and the quantification results for specific regions are shown in Supplemental Figures 4–6. While similar numbers of DAPI-labeled cells were quantified, fluorescence imaging revealed that many cells in the spinal cord had strong EB signal intensity in saline-injected SCI animals. In contrast, MHC1 sham-operated animals had no signal. After SCI, significantly fewer cells were labeled with EB in MHC1-treated mice (Figure 3, B and C). Altogether, these results indicate that the MHC1 antibody was delivered to the injury site of the spinal cord after SCI and that the presence of MHC1 resulted in the significant reduction in the numbers of cells with active hemichannels.

### Acute administration of MHC1 after SCI results in hind limb function improvement, gliosis and lesion size reduction, and neuronal protection.

We used 2 models to assess the efficacies of MHC1 on recovery after partial SCI. For each model, we measured hind limb function using the Basso Mouse Scale (BMS) (29, 30). For model 1, we delivered a contusion directly to the spinal cord with a broad impactor tip, resulting in a slightly broader and diffuse injury. For model 2, we subjected the spinal cord to a specific amount of pressure in dynes to create a more focused injury. We also used a third model (model 3), a forceps compression model that caused a complete SCI and so had no opportunity for full recovery of hind limb function. MHC1 administration improved outcome (functional or via other measures) from SCI in all 3 models.

**Figure 4:**
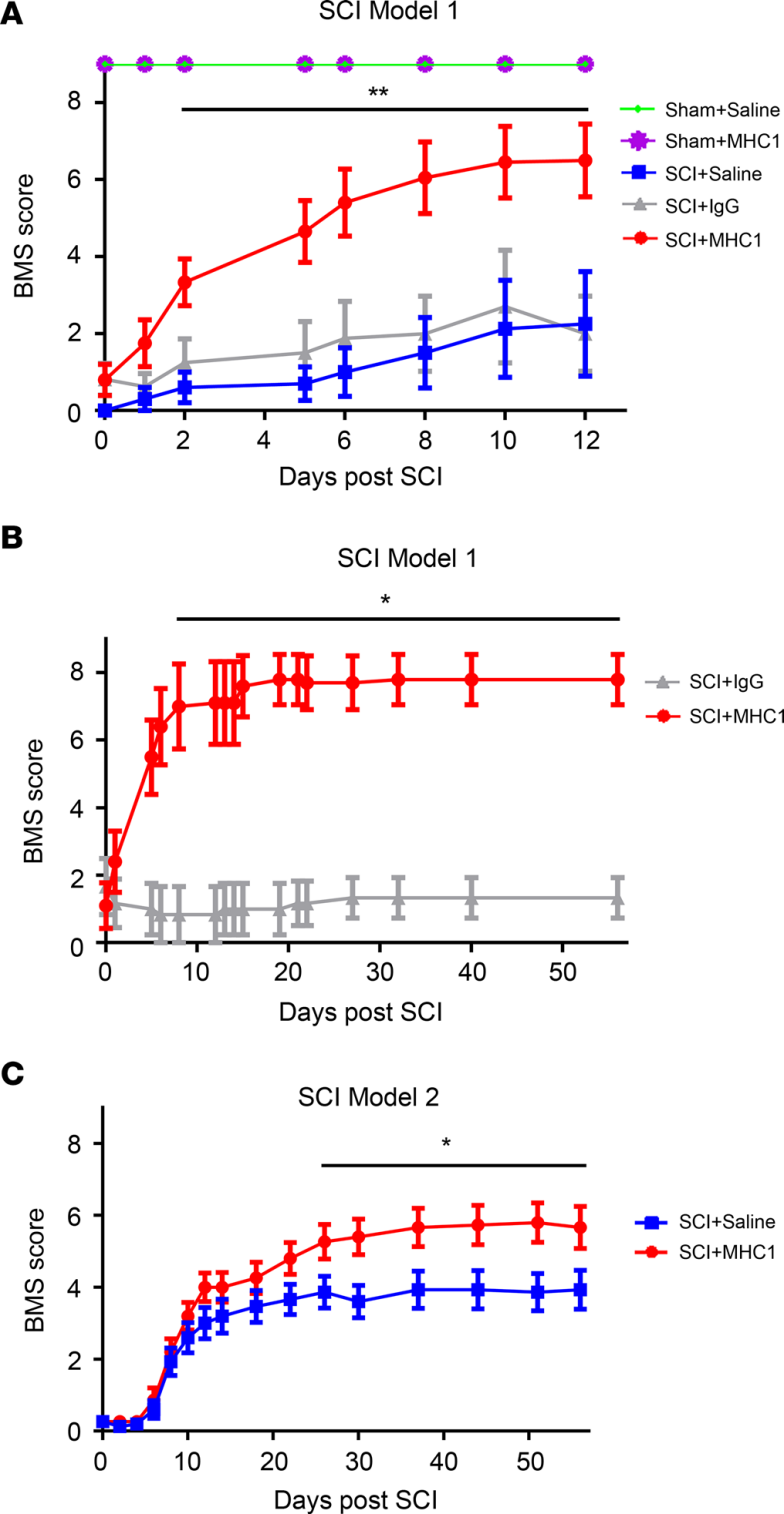
Mice with SCI recover hind limb function after treatment with Cx43 antibody. After SCI, the hind limb function was evaluated with BMS, 1–9. 0 = no hind limb function and 9 = completely normal hind limb function. (**A**) Mice were subjected to broad impactor of SCI (model 1), and BMS score was measured for 12 days. MHC1 antibody–treated SCI mice were compared with IgG-treated SCI mice. Sham+Saline (*n* = 5), Sham+MHC1 (*n* = 5), SCI+Saline (*n* = 5), SCI+IgG (*n* = 8), SCI+MHC1 (*n* = 10). (**B**) Mice were subjected to broad impactor of SCI (model 1), and BMS score was measured for 56 days. SCI+IgG (*n* = 3), SCI+MHC1 (*n* = 5). (**C**) Mice were subjected to focused impactor of SCI (model 2), and BMS score was measured for 56 days. SCI+Saline (*n* = 15), SCI+MHC1 (*n* = 15). The data are presented as mean ± SEM. Statistical analysis consisted of linear mixed model of repeated measures, with antedependent covariance structure of time in days as best fit model (**A**); linear mixed model of repeated measures, with autoregressive covariance structure of time as best fit model (**B**); and 2-way repeated measures ANOVA with time as best fit model (**C**). *P < 0.05, **P < 0.01.

In sham-operated mice, the BMS remained at 9, indicating normal hind limb function. In SCI mice, BMS scores exceeding 3 at 5.5 hours after the injury suggested that the contusion injury was very mild and would have a full recovery later. Therefore, we excluded the mice retaining such levels of hind limb function in saline-, IgG-, and MHC1-treated groups. In all nonexcluded mice subject to SCI, BMS scores were statistically similar, between 0 and 3. After the 5.5-hour time point, SCI mice treated with MHC1 showed a significantly greater improvement in hind limb function within the first 12 days (Figure 4A) and significantly greater improvement in hind limb function for up to 8 weeks compared with mice treated with saline or nonspecific IgG (Figure 4B for model 1 and Figure 4C for model 2). In the broad impact model 1, mice treated with MHC1 antibody showed near-immediate, significant improvement beginning with day 2 after SCI (Figure 4A), with continual and gradual improvement over the remainder of the assessment period (Figure 4B). The control groups (treated with IgG) showed much less improvement than those treated with MHC1 (Figure 4, A and B). Moreover, IgG controls did not exhibit any detrimental functional effects compared with saline-treated controls up to 12 days postinjury (Figure 4A). This result indicates that immunotherapy per se is not detrimental in mice. In SCI induced by the model 2 focused impact, which used stronger, more finely pointed force than that used for model 1, both groups showed improvements in the BMS score starting from day 6 to day 56. MHC1-treated animals displayed a greater improvement of hind limb function than controls, and significant differences were observed from day 26 to the end of the assessment period (day 56) (Figure 4C). Altogether, the data from the partial SCI models suggest that treatment with the MHC1 antibody elicits significant improvement of hind limb function after acute SCI.

**Figure 5:**
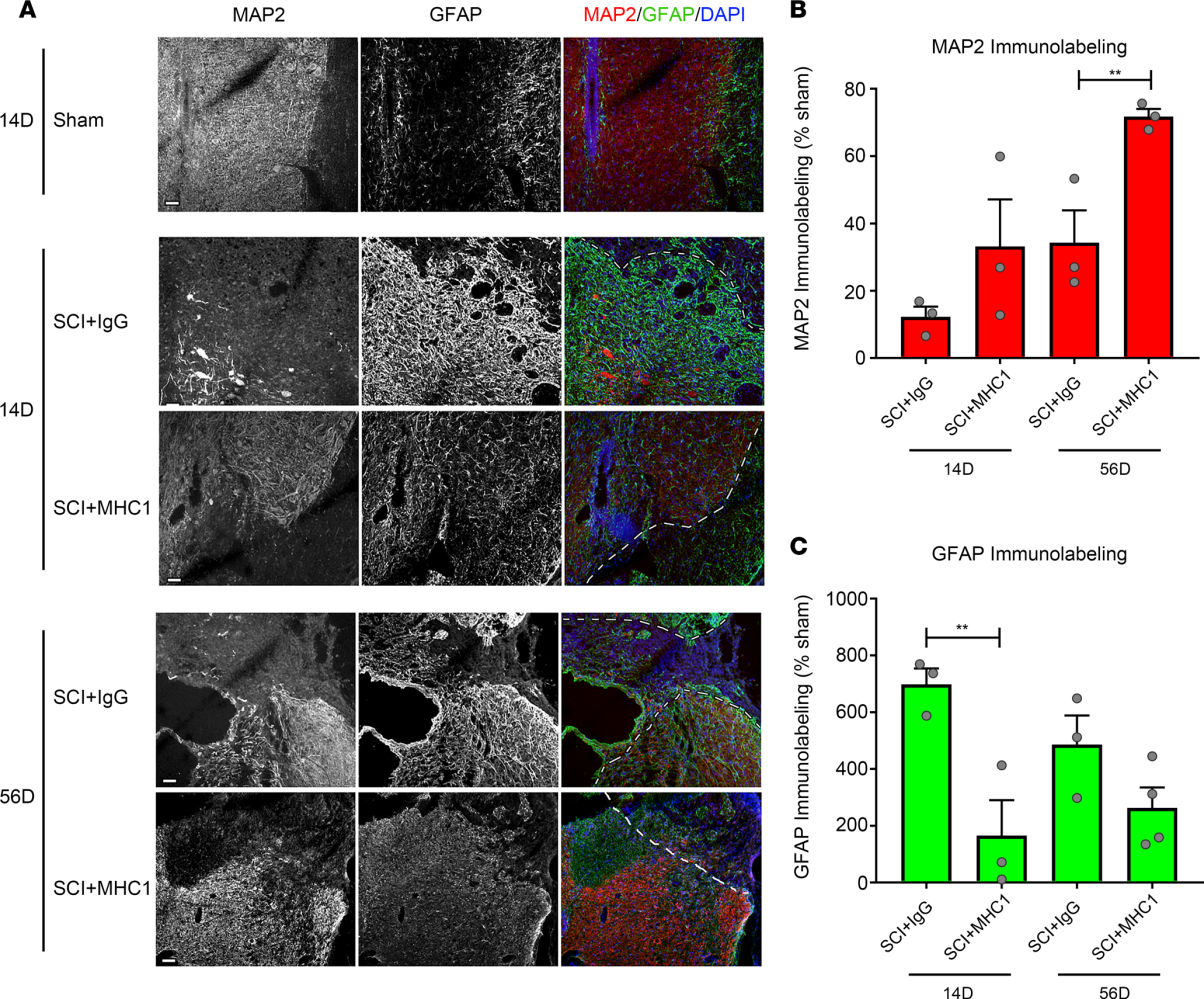
Blocking Cx43 hemichannel function by MHC1 antibody decreases spinal cord gliosis and protects neuron survival under the model 1 impact. Spinal cords were isolated 14 days or 56 days after the model 1 impact of SCI treated with IgG control or MHC1 antibody. (**A**) The frozen sections were immunolabeled with anti-MAP2 or anti-GFAP antibody and counterstained with DAPI. The representative images of MAP2 and GFAP immunofluorescence were shown from the perilesion areas. (**B**) The quantification of MAP2-positive signals by NIH ImageJ software. SCI+IgG 14D (*n* = 3), SCI+MHC1 14D (*n* = 3), SCI+IgG 56D (*n* = 3), SCI+MHC1 56D (*n* = 3). (**C**) The quantification of GFAP-positive signals by NIH ImageJ software. SCI+IgG 14D (*n* = 3), SCI+MHC1 14D (*n* = 3), SCI+IgG 56D (*n* = 3), SCI+MHC1 56D (*n* = 4). All images were taken from perilesional area, which is confined to 1.5 mm from injury border of sagittal sections. The white dotted lines label the lesion border. Scale bar: 50 μm. Data are presented as mean ± SEM. Unpaired *t* test (1 tailed) was used in statistical analysis. **P < 0.01.

**Figure 6:**
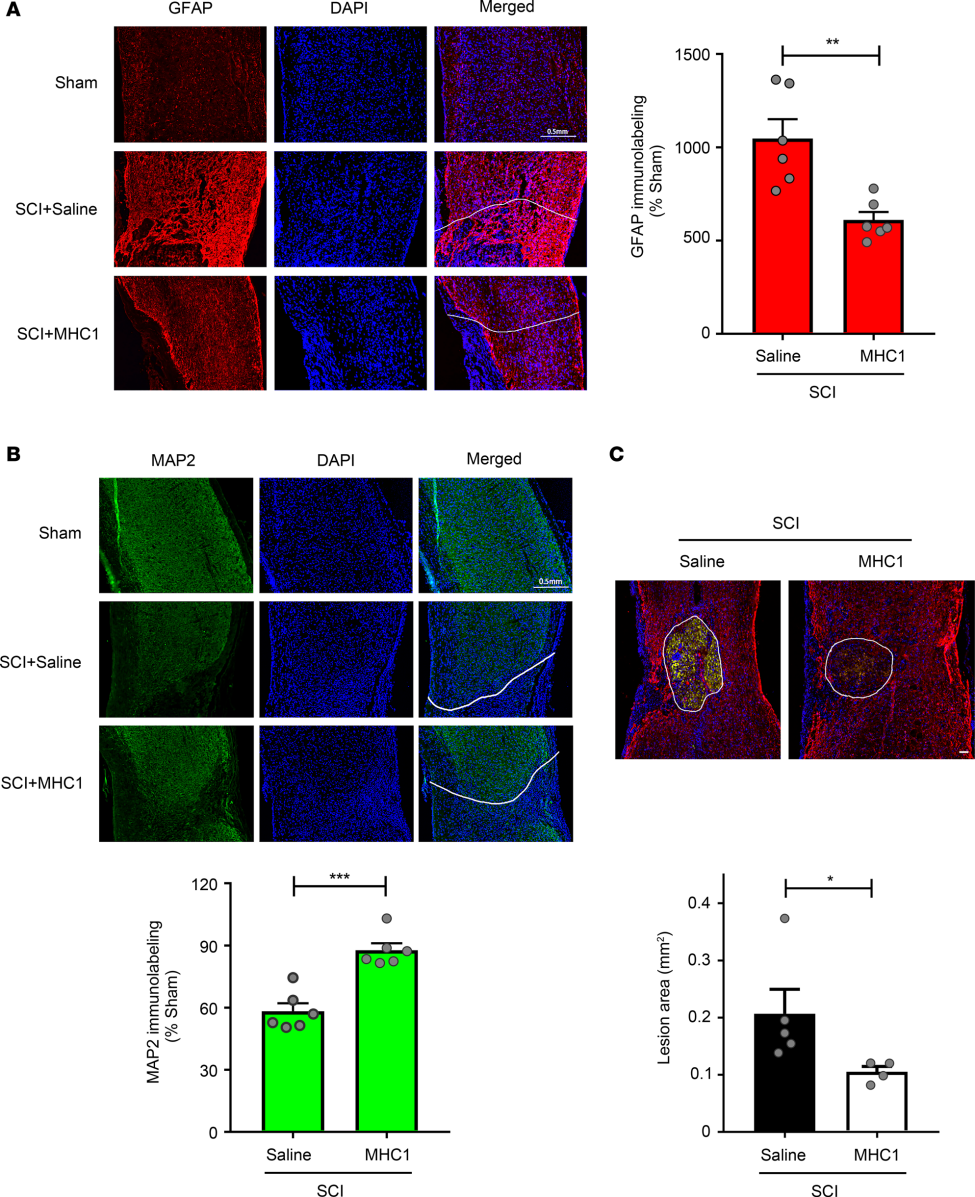
Blocking Cx43 hemichannel function by MHC1 antibody decreases spinal cord gliosis, protects neuron survival and reduces SCI lesion under the model 2 impact. Spinal cords of SCI treated with saline control or MHC1 antibody were isolated 56 days after the model 2 impact. (**A**) The frozen sections were immunolabeled with anti-GFAP antibody and counterstained with DAPI. Left panels show representative images of GFAP immunofluorescence. Right panel show the quantification of GFAP-positive signals from perilesional area (area < 0.5 mm from injury border on sagittal sections) by NIH ImageJ software. Images were taken from perilesional area. White line labels the injury site border, and the area under the white lines is the lesion site. Scale bar: 0.5 mm. (**B**) The frozen sections were immunolabeled with anti-MAP2 antibody and counterstained with DAPI. Upper panels show representative images of MAP2 immunofluorescence, and lower panel shows the quantification of MAP2-positive signals from perilesional area (area < 0.5 mm from injury border on sagittal sections) by NIH ImageJ software. All images were taken from perilesional area. White line labels the injury site border, and the area under the white lines is the lesion site. Scale bar: 0.5 mm. (**C**) The white lines define SCI lesion areas (upper panel), and the sizes of lesion areas were quantified by NIH ImageJ software (lower panel). Scale bar: 50 μm. Data are presented as mean ± SEM. Unpaired *t* test (1 tailed) was used in statistical analysis. (**A**–**C**). For GFAP and MAP2 staining, SCI+Saline (*n* = 6), SCI+MHC1 (*n* = 6). For lesion area, SCI+Saline (*n* = 5), SCI+MHC1 (*n* = 4). *P < 0.05, **P < 0.01, ***P < 0.001.

We next examined the effect of MHC1 on neuronal survival as indicated by expression of neuronal marker microtubule-associated protein 2 (MAP2). Expression of MAP2 in the perilesional area (0.5 mm from the lesion site border) was significantly lower in IgG-treated mice 14 days and 56 days after SCI with the model 1 broad impact than in sham-operated mice, indicating loss of neurons in the perilesional area. In MHC1-treated mice, the MAP2 signal was substantially greater compared with IgG-treated mice (Figure 5A). Quantitative analysis of MAP2 immunolabeling showed that MHC1 treatment significantly preserved MAP2 expression 14 and 56 days after SCI (*F* = 21.31 and *F* = 18.13 on 14- and 56-day experiments, respectively) (Figure 5B). Similar results were obtained 56 days after SCI with the model 2 focused impact (*F* = 1.338) (Figure 6B). These results suggest that MHC1 treatment reduces neuronal loss after SCI.

We evaluated the effect of MHC1 on reactive astrogliosis after SCI by measuring the expression of GFAP, a marker for reactive astroglia and glial scars. Expression of GFAP was markedly higher in the perilesional area of saline-treated mice after SCI with the model 1 broad impact (Figure 5A). MHC1 treatment caused significantly attenuated GFAP immunolabeling at 14 and 56 days after SCI (*F* = 5.03 and *F* = 1.497 in 14- and 56-day experiments, respectively) (Figure 5C). Similar results were obtained 56 days after SCI with the model 2 focused impact (*F* = 5.763) (Figure 6A). Moreover, we tested the effect on gliosis in the model 3 mice subjected to a complete SCI. Even though mice did not exhibit functional recovery in this model, a significant reduction in gliosis compared with SCI controls was evident (Supplemental Figure 7). The significant reduction of GFAP expression in the peritraumatic areas of all 3 SCI models suggests that MHC1 plays an important role in inhibiting astrogliosis and glial scar formation after SCI.

**Figure 7:**
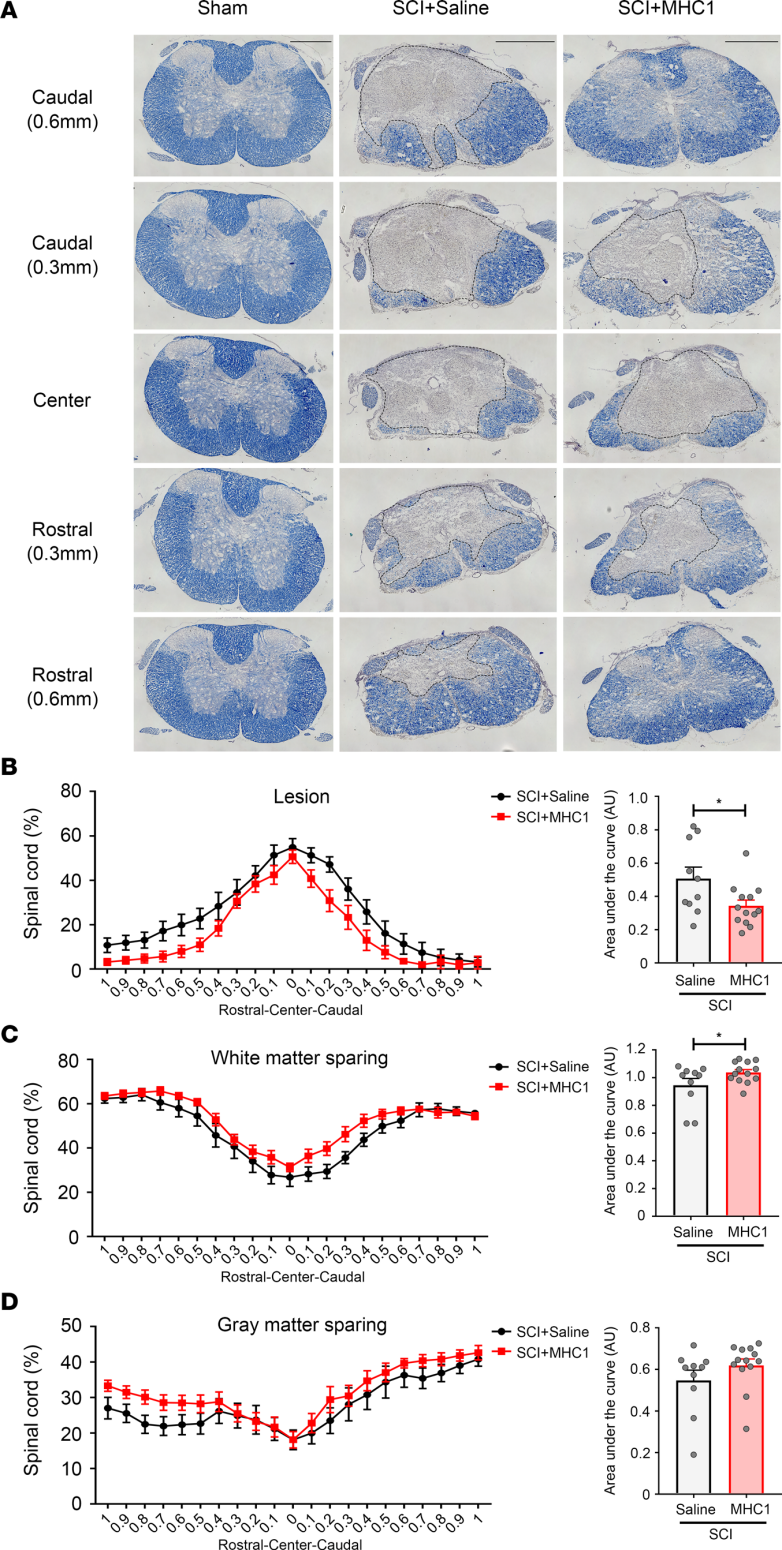
Blocking Cx43 hemichannel function by MHC1 antibody reduces SCI lesion and increases white matter and gray matter sparing under the model 2 impact. Spinal cords were isolated 30 days after the model 2 impact of SCI with the treatment of saline control or MHC1 antibody. The 10 μm thickness cryo-tissue sections were collected every 100 μm for a total 2 mm length centered on injury site, stained with Eriochrome cyanine R (**A**), and analyzed for lesion (**B**), white matter sparing (**C**), and gray matter sparing (**D**) by FIJI-ImageJ. The black dotted lines label the lesion border. Area under the curve was calculated. SCI+Saline (*n* = 10), SCI+MHC1 (*n* = 13). Scale bar: 500 μm. Data are presented as mean ± SEM. Unpaired *t* test (1 tailed) was used in statistical analysis. *P < 0.05.

Reduced astrogliosis could be the result of a decreased lesion size, rather than an attenuation of mechanisms that promote glial scar formation regardless of lesion size. To test whether the overall lesion size was affected by MHC1 treatment, we measured the lesion size 56 days after SCI with the model 2 focused impact. We observed a significant reduction of SCI lesion sizes in mice treated with MHC1 (*F* = 26.53) (Figure 6C). To further test the effect on tissue preservation, we assessed spinal cord histopathology 30 days after SCI with the model 2 focused impact using Eriochrome cyanine R (ECR) staining for myelin (Figure 7A). We harvested 10 μm serial transverse sections for a total of 2 mm surrounding the injury site and stained with ECR. We measured ECR staining in contiguous 100 μm bins (Figure 7, B–D). The spared white matter was defined as the areas that were stained for ECR, whereas gray matter remained with stereotypic light ECR color and consistent neuropil texture containing neuronal and glial cell bodies, and the lesion was stained in light gray color with disrupted tissue (31). Lesion size was measured as the disrupted area of both these regions centered by the area in the section with the greatest lesion area. The serial transverse sections showed significantly decreased lesion size in MHC1-treated mice compared with saline-treated mice (*F* = 2.962) (Figure 7, A and B). MHC1 treatment also induced significant white matter (*F* = 4.309) sparing and a trend of gray matter sparing (*F* = 1.943) in perilesional area (Figure 7, C and D). These data suggest that MHC1 treatment significantly reduces the lesion size and increases the abundance of white and gray matter spared after SCI.

### Minimal improvement of limb function and gliosis by late-onset treatment of MHC1 after SCI.

**Figure 8:**
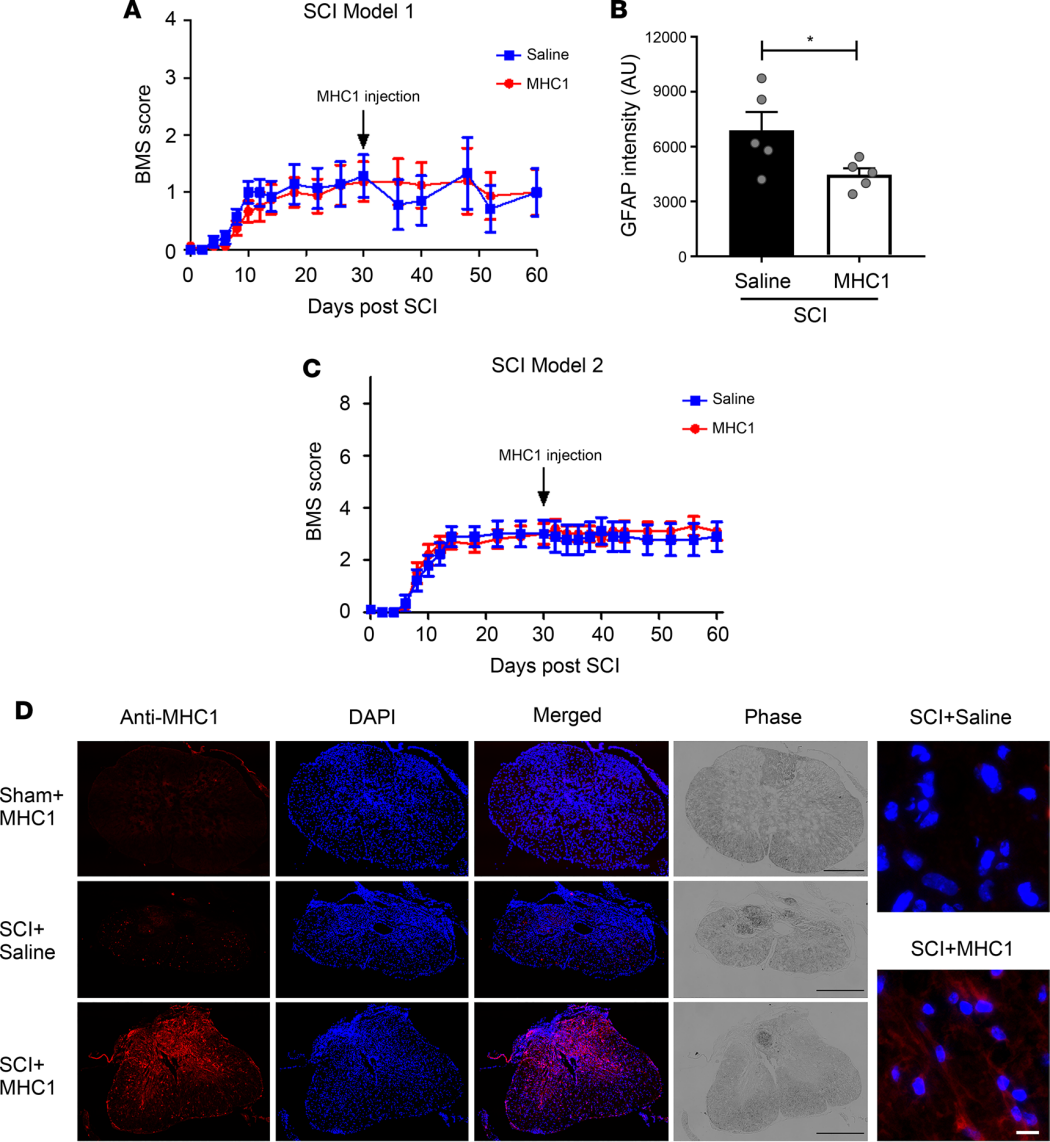
Mice do not show improved recovery with Cx43 antibody 1 month after SCI. Thirty days after SCI, mice were injected with 25 mg/kg MHC1 via intracerebroventricular route. (**A**) Mice were subjected to broad impact (model 1). BMS test was performed 30 days after MHC1 administration. SCI+Saline (*n* = 14), SCI+MHC1 (*n* = 16). (**B**) Mice were subjected to a broad impact (model 1). The frozen sections of spinal cords 60 days after SCI were immunolabeled with anti-GFAP antibody, and GFAP-positive signals from perilesional area (area < 0.5 mm from injury border on sagittal sections) were quantified using NIH ImageJ software. Data are presented as mean ± SEM. SCI+Saline (*n* = 5), and SCI+MHC1 (*n* = 5). (**C**) Mice were subjected to focused impact (model 2), and BMS score was determined in mice treated with MHC1 or saline control. SCI+Saline (*n* = 9), SCI+MHC1 (*n* = 10). (**D**) Mice were subjected to focused impact (model 2). Four hours after ICV injection with saline or MHC1, spinal cords were isolated; the frozen sections of spinal cords around perilesional sites were immunolabeled with anti-human IgG secondary antibody and counterstained with DAPI. Scale bar, 500 μm (black), 10 μm (white, right panels). Two-way repeated measures ANOVA with time was used for BMS statistical analysis (**A** and **C**). Unpaired *t* test (1 tailed) was used in statistical analysis (**B**). *P < 0.05.

We tested whether administration of MHC1 outside of the acute period after injury would prove beneficial post-SCI. The BSCB is reported to be closed 2 weeks after SCI in mice (26, 32). Therefore, we performed intracranial injections of MHC1 to administer antibody within the cerebrospinal fluid. We injected MHC1 or saline through intracerebral ventricular (ICV) injection 30 days after SCI using the model 1 broad impact and observed mice for another 30 days. Both saline and MHC1 groups showed some improvements for BMS scores of hind limb function from day 8 to day 60. However, there was no difference between the 2 groups (Figure 8A). This result indicates that the MHC1 administered outside a 30-day window fails to improve hind limb function over controls. We similarly measured gliosis and found a significant difference in GFAP expression between these 2 groups (*F* = 7.984) (Figure 8B). It is plausible that the reduced glia scar might not allow enough axon regeneration, and thus ICV MHC1 has no effect on motor performance. We also confirmed the results of hind limb function using the model 2 focused impact (Figure 8C). To test whether these results might be explained by an inability of MHC1 to penetrate the spinal cord via cerebrospinal fluid circulation after ICV injection, anti-human IgG antibody was used to detect MHC1 in the injury region 30 days after SCI with the model 2 focused impact (Figure 8D). Immunolabeling with anti-human IgG antibody showed positive signals in MHC1-injected samples, indicating the presence of MHC1 around the lesion region (Figure 8D). These data support the conclusion that MHC1 can reach the lesion via ICV injection, but administration is efficacious only within a specific time window after SCI.

## Discussion

Cx43 is abundantly expressed in astrocytes in the CNS, forming gap junctions and hemichannels. Cx43 hemichannels are known to be involved in CNS inflammation (33, 34). Previous studies reported that astrocyte-specific Cx43-conditional knockout (Cx43-cKO) mice exhibit partial resistance to SCI, and when compared with WT mice, have higher BMS numbers (13). These results suggest that Cx43 plays a negative role in the recovery after SCI. Additionally, astrocyte-specific Cx43-cKO mice have attenuated posttraumatic release of ATP from astrocytes (13). This study indicates the potential involvement of Cx43 hemichannels, which have been reported to mediate the release of ATP from astrocytes, leading to microglial recruitment and neuronal cell death (35–38). Cx43-deficient mice could have compromised function of both types of channels formed by Cx43, gap junction channels and hemichannels. Currently there is no animal model available with which we could distinguish a deficiency in gap junction channels from a deficiency in hemichannels. In this study, we reported development of a potentially novel monoclonal antibody that exhibits a specific inhibitory effect on Cx43 hemichannels but had no effect on gap junction channels. We showed that acute administration of this hemichannel-blocking antibody significantly improved neuronal recovery after traumatic SCI with reduced glial scar formation, reduced lesion sizes, and improved motor motion recovery, thereby implicating hemichannels as playing a significant role in secondary expansion of SCI.

We modified 2 spinal cord contusion mouse models from previously published studies using the C57BL/6 mouse strain (29, 30). These 2 models closely recapitulate partial SCI occurring in most patients with partial paralysis and physical impairment symptoms. With both models, we observed a partial recovery in the nontreated control groups. However, during the 8 weeks postinjury, the antibody-treated group showed significantly greater recovery of hind limb function (Figure 4). Interestingly, there was little or no recovery for the first 5 days for the model 2 focused SCI injury, unlike the model 1 broad injury, which showed recovery at an earlier time. The model 2 impact is pointed and focused, unlike model 1 with a broader impact. The underlying injuries between these 2 models are expected to be dissimilar enough to explain the differences in recovery; we expect that the model 2 focused injury could be more severe than the model 1 broad injury.

The intrinsic capacity of the spinal cord to repair wounds may fail due to inflammation, formation of scar tissue, and other factors. Our results suggest that inhibition of Cx43 hemichannels decreases scar formation and improves recovery. We hypothesize that this recovery is driven by reduced release of proinflammatory and neurotoxic factors, such as ATP and glutamate (13, 39–41). Moreover, MHC1 preserved white and gray matter. In contrast to results observed with partial injury, we did not observe any functional recovery after antibody administration following complete injury of the spinal cord to induce total paralysis in our model 3 complete injury. However, significant reduction of gliosis was observed in the complete SCI model (Supplemental Figure 7). This evidence suggests that inhibition of astrocyte Cx43 hemichannels improves SCI recovery through preservation of neurons rather than through neuronal repair or regeneration.

Cx43 hemichannels in astrocytes are mostly closed under normal physiological conditions. Under acute SCI, Cx43 hemichannels are open, and this opening is likely mediated by cytokines, such as IL-1β (42). In vitro studies have shown that proinflammatory factors, including IL-1β and TNF-α, and mechanical stimulation induce opening of Cx43 hemichannels in astrocytes (21, 43–46). In agreement with results of studies on Cx43 hemichannel function in brain astrocytes, our results show that IL-1β induces the activation of Cx43 hemichannels in primary astrocytes isolated from spinal cords. We further validated hemichannel activity using a polyclonal Cx43 hemichannel-blocking antibody we developed earlier (24). The opening of hemichannels permits release (effluxes) of inflammatory factors and neurotransmitters including ATP and glutamate. These factors further evoke inflammatory responses and promote glial scar formation and neuronal death (13, 39–41). This process is called “secondary injury.” In addition to IL-1β, we observed that mechanical stress, mimicking the physical trauma experienced by spinal cords, activates hemichannels. For the first time to our knowledge, we also demonstrated the activation of Cx43 hemichannels in the spinal cord in response to SCI in vivo. We propose that SCI evokes an initial inflammatory response with increased IL-1β release. Elevated levels of IL-1β and mechanical stress cause the opening of Cx43 hemichannels and release of proinflammatory and excitotoxic factors, such as ATP and glutamate. These factors promote expansion of the secondary injury, eventually causing increased neuronal cell death and glial scar formation. To assess the potential involvement of other channels in response to MHCI, such as pannexin channels, which form hemichannels on the cell surface similar to connexins, we found that MHC1 had no effect on opening of pannexin channels induced by ATP in cells that did not express Cx43 (Supplemental Figure 2). While we showed that MHC1 does not affect pannexins, this study cannot exclude the effect of pannexins or other mediators that could associate with Cx43 hemichannels in SCI pathology.

Previous studies showed that the BSCB is permeable up to 2 weeks after SCI in mice (26, 32). To determine the efficacy of the antibody after secondary injury expansion has occurred, we administered the antibody through ICV injection at 30 days after SCI. Although MHC1 was detected after ICV delivery, it had no effect on recovery. One possible explanation for the failure of MHC1 to be beneficial at this later stage of injury is that in contrast to acute SCI, the levels of proinflammatory factors, including IL-1β, TNF-α, and other interleukins, are significantly reduced by 24 hours after SCI and undetectable 1 week after SCI (47–49). This evidence suggests that the hemichannels are no longer pathologically open at the later stages of injury because of a lack of proinflammatory stimulus and ongoing trauma. Moreover, the acute secondary expansion of the injury will likely spread to a broad area, resulting in maximal glial scarring and acute neuronal loss in the tissue during postinjury homeostasis. A previous study (50) showed that in a rat model of SCI, CBX, the nonspecific chemical connexin channel blocker, only had an effect in the acute phase, but not in the chronic phase, of SCI. Based on these results, we hypothesize that MHC1 is only efficacious during the early acute phase of SCI, when hemichannels are pathologically open.

The dual role of the glial scar in inhibiting neuronal growth or promoting recovery after injury is debatable and an important aspect of the postinjury microenvironment (51). However, significant glial scarring can itself lead to comorbidities associated with SCI, including allodynia and hyperalgesia (52–54). We observed a great reduction of scar formation in the antibody-treated group indicated by a decrease in GFAP signal. This decrease is consistently correlated with the reduction of neuron loss indicated by MAP2 staining of axons. Our data do not indicate whether the reduced gliosis prevented the spread of secondary injury per se. We hypothesize that reduced gliosis is merely an indicator of reduced secondary injury in the spinal cord after MHC1 administration, rather than a direct effect of MHC1 on cellular mechanisms that regulate gliosis. A reduction in secondary injury due to MHC1 is expected to cause reduced numbers of activated astrocytes in the perilesional area, but we expect those astrocytes likely retain the ability to become activated in response to stressful stimuli.

Our data, as well as data from previous published studies, suggest that Cx43 hemichannels are a potential target for drug development for SCI. It has been shown that a Cx43 mimic peptide, Peptide5, reduces inflammation and tissue damage and improves functional recovery after SCI (4, 10). However, this peptide inhibits hemichannels and can block gap junctions as well (4), similar to other Cx43 mimic peptides (55). Cx43 has a fast turnover time, around 3 hours (56). Cx43 forms gap junctions in a closed configuration, which requires appropriate cadherin/catenin-mediated cell adhesion at regions of cell adhesion or near tight junctions. Hemichannels, however, often locate on the free cell surface of astrocytes to facilitate exchange of molecules between the inside and outside of the cell. Therefore, MHC1 antibody can readily bind to Cx43 hemichannels on the free surface but not gap junctions with apposing extracellular domains located in a tight extracellular space. As expected, MHC1, possibly due to its bulky size and inability to access the closely associated space where gap junctions are located, does not inhibit gap junctions but has a strong effect on hemichannels. Therefore, treatment with this hemichannel-blocking antibody at the acute phase of SCI results in effectively reducing inflammatory responses, reducing lesion size, and minimizing scar formation and neuronal death. This study shows a great potential for the development of a new line of antibody therapeutics for SCI.

## Methods

### Materials.

HeLa-Cx43, a cell line stably transfected with mouse Cx43, was provided by Bruce Nicholson at the University of Texas Health Science Center at San Antonio (UTHSCSA). Chicken anti-GFAP (AB5541) and rabbit anti-GFAP (AB5804) antibodies were obtained from MilliporeSigma. Chicken anti-MAP2 antibody was obtained from Abcam (ab5392). All other reagents were obtained from MilliporeSigma or Thermo Fisher Scientific. Astrocyte medium was prepared as 10% fetal calf serum in DMEM/F12 with Primocin (Thermo Fisher Scientific).

### Generation of the MHC1 monoclonal antibody.

The hybridoma cell lines were generated by Abmart. Briefly, mice were immunized with a Cx43 extracellular domain peptide intraperitoneally with 50 μg of the peptide in complete Freund’s adjuvant and then boosted repeatedly with the peptide antigen formulated in incomplete Freund’s adjuvant. Spleens were harvested and fusions performed as previously described (57, 58). After functional characterization of the hybridoma clones, genes that encode the antibody heavy and light chain variable regions were cloned from the mouse hybridoma cell line HC1 by reverse transcription PCR, using a combination of a group of cloning PCR primers. Two rounds of PCR were performed by incorporating overlapping sequences at the 3′- and 5′-ends, allowing infusion cloning of the variable regions into vectors for expression of human constant regions of heavy and light chains based on a protocol as described previously (59). The resultant mouse-human chimeric MHC1 antibody was cloned from mouse hybridoma cell lines targeting extracellular domains of Cx43. Heavy and light chain constructs were cotransfected into human embryonic kidney freestyle 293 (HEK293F) cells (Thermo Fisher Scientific) using transfection reagent PEI (MilliporeSigma). After 7 days of expression, supernatants were harvested and antibodies were purified by affinity chromatography using protein A resin as we reported before (60).

### Affinity measurement with ELISA and SRP-Octet RED96.

For antibody affinity measurement using ELISA, the biotinylated peptides were synthesized by WuXi Biologics. A 50 μL solution containing 20 μM Cx43 peptides or control peptides in PBS and 0.1% BSA was used to coat a well of a streptavidin plate (Thermo Fisher Scientific). After 3 washes with PBS, serial dilutions of MHC1 antibody (1 × 10^–5^ to 1 μM) were added to the plate and incubated for 2 hours. Human IgG was used as a control for MHC1. After 3 washes, LI-COR IR Dye–labeled goat anti-human IgG antibodies (LI-COR catalog 925-32232) were added to each well and incubated for 1 hour. The plate was thoroughly washed and scanned with Odyssey infrared imaging system (LI-COR). Data were analyzed and plotted with GraphPad Prism software. EC_50_ value was determined with best fit (61, 62).

For antibody affinity measurements using Octet RED96, MHC1 antibody (133.4 μM) was loaded onto the protein G biosensors for 4 minutes. Following a short baseline in kinetics buffer, the loaded biosensors were exposed to a series of Bio-mab-M1 concentrations (0–500 nM), and background subtraction was used to correct for sensor drifting. All experiments were performed with shaking at 1000 rpm. Background wavelength shifts were measured from reference biosensors that were loaded only with antibody. ForteBio’s data analysis software was used to fit the data to a 1:1 binding model to extract an association rate and dissociation rate. The *K_D_* was calculated using the ratio k_off_/k_on_.

### Isolation of primary spinal cord astrocytes.

The acquisition of spinal cord astrocytes was based on published protocols (23, 63). Briefly, adult male mice (8–12 weeks old, C57BL/6, from The Jackson Laboratory) were humanely sacrificed using isoflurane. Fresh spinal cord was dissected from the thoracic and lumbar regions, excluding the dorsal root ganglion. After washing in sterile, cold PBS, tissues were finely diced into 0.5–1 mm diameter pieces and incubated in 3 mL trypsin-EDTA (0.25%, Thermo Fisher Scientific) in a cell culture incubator (5% CO_2_, 37°C) for 20 minutes. Trypsin was neutralized with astrocyte medium (3 mL) and centrifuged (1000*g*, 3 minutes). The pellet was resuspended in 6 mL astrocyte medium and serially homogenized first in a 10 mL serological pipette (approximately 10×) and then in a glass Pasteur pipette (approximately 10×) until most of the tissue was broken up and the mixture was cloudy. The tissue homogenate was filtered through a 40 μm sterile mess strainer, then was transferred to a T75 cell culture dish with 10 mL astrocyte medium. The suspension was incubated overnight for 24 hours. The cell suspension was transferred to a new T75 dish for a subsequent overnight incubation. Each dish was washed once with cold PBS, then refed with warm astrocyte medium. Culture medium was changed weekly while astrocytes grew to confluence. Astrocytes were used for experiments before passage 5.

### Animals and SCI models.

Mice were housed in a temperature-controlled room with a 12-hour light/12-hour dark cycle at UTHSCSA Institutional Lab Animal Research facility, under specific pathogen–free conditions. All described animal protocols were reviewed and approved by UTHSCSA Institutional Animal Care and Use Committee (IACUC), in accordance with policies dictated by the Office of Animal Welfare at the NIH, USA. Ten-week-old male C57BL/6 mice from The Jackson Laboratory were used in the study. Animals were anesthetized with isoflurane (1% in oxygen), and the surgical site was depilated and cleaned prior to making an incision in the midline of skin over the thoracic vertebrae. The muscle and tissue overlying the lamina were sharply dissected to expose lamina, and laminectomy was performed at the T9–11 vertebral level with angled scissors. The vertebral column was secured using a stereotaxic frame. A contusion injury was induced on the exposed cord using 2 SCI models. For model 1, broad impact was introduced using TBI-0310 Impactor (Precision Systems and Instrumentation) with a mouse impacting tip (1.9 mm diameter) for a 3.0 m/s, 1500 ms, 2 mm injury. For model 2, focused impact was introduced using Infinite Horizon IH-0400 Impactor (Precision Systems and Instrumentation) with a mouse impacting tip (1.25 mm diameter) and an impact force of 70 kdynes. The impacts were implemented on the midline of exposed spinal cord in both models to cause bilateral paralysis. The muscle and skin openings were closed with 5-0 absorbable suture. MHC1 antibody (25 mg/kg) or saline was administered by intraperitoneal injection 30 minutes after injury. After surgery, buprenorphine (0.05 μg/g s.c.) was administered twice a day for 2 days. Manual bladder expression was performed twice a day until mice recovered bladder expression (29). The sham-operated control group received the same surgical procedure without the contusion injury.

### In vivo antibody localization and EB uptake assays to assess antibody delivery and hemichannel function, respectively.

The delivery of antibody to spinal cord location was assayed by injecting MHC1 (25 mg/kg), a human anti-human congenital human cytomegalovirus IgG isotype control (25 mg/kg), or saline intraperitoneally at 30 minutes after SCI under model 2 that has a focused impact. The delivery of antibody to spinal cord at 30 days after SCI was assayed by injecting MHC1 (25 mg/kg) or saline through ICV injection under model 2 that has a focused impact. Four hours after injection, mice were sacrificed, and the spinal cords were harvested. Dye uptake was used to study hemichannel opening under SCI model 1 that has a broad impact. EB dye (MW: 900 Da) (200 mg/kg) and FITC-dextran (MW: 10,000 Da) (200 mg/kg) were coinjected 4 hours post-MHC1 treatment via the tail vein. One hour after administration of the dye, mice were anesthetized, and cardiac perfusion was performed with cold PBS and 4% paraformaldehyde (PFA). Spinal cords were isolated and prepared for frozen immunohistochemistry by incubation in 4% PFA overnight and then 30% sucrose/PBS for 3 days. Tissue was frozen in optimal cutting temperature compound (Thermo Fisher Scientific) using a methyl-butane bath and liquid nitrogen. Then, 10 μm sections were taken for subsequent analysis.

### Behavioral studies.

Following spinal cord contusion injury, each mouse was assessed for hind limb functional recovery with an open-field testing paradigm. Animals were tested at days 0, 2, 4, 6, 8, 10, 12, 14, 18, 22, 26, 30, 37, 44, 51, and 56 of the postoperative recovery period. Hind limb locomotor function was assessed using the BMS (64). BMS scores were assessed by 2 independent operators who were blinded to the groups.

### ICV injection.

One month after SCI, the mice were anesthetized with isoﬂurane and placed in a stereotactic frame. The skull was exposed, and a 1 mm diameter hole was drilled in the skull with coordinates anteroposterior: –0.2 mm, mediolateral: 1 mm, dorsoventral: –2.5 mm according to bregma. Then, 2 mg (2 μL) or 14.6 mg MHC1 (3.5 μL) was injected into the lateral ventricle using a Hamilton 701N syringe connected with an Integrated Stereotaxic injector (Stoelting) (65).

### Mechanical stimulation by Flexcell stretching.

Primary spinal cord astrocytes isolated from mouse spinal cords were cultured in Flexcell 6-well plates (Flexcell International) coated with laminin and poly-d-lysine. Astrocytes were grown to 80% confluence, and astrocyte medium was replaced with fresh medium before experimentation. For both treated and nontreated plates, wells were incubated with vehicle, Cx43E2 antibody (24) (20 nM), or MHC1 (66.7 nM). The plates were incubated for 30 minutes and then were subjected to mechanical strain with Flexercell Strain Unit (model FX-2000) with settings –6.3 kPa, 30 cycles/min, and 600 cycles in total. After treatment, 50 μM of EtBr was added to each well and incubated for 5 minutes. Following 3 rinses with PBS, cells were fixed with 2% PFA for 10 minutes.

### Cell culture, EtBr dye uptake assay, and scrape-loading dye coupling assay.

Parental HeLa cells and HeLa-Cx43 cells were cultured in DMEM with 10% FBS. Human cortical or mouse spinal cord astrocyte cells were incubated with DMEM/F12 medium plus 10% FBS until confluence. After cells reached 70%–80% confluence, the cells were preincubated with 66.7 nM MHC1, 66.7 nM IgG, 6.7 nM Cx43E2, or 100 μM CBX for 30 minutes. The medium was then removed and cells were incubated with HCO_3_–free saline medium with 10 mM HEPES, 154 mM NaCl, 5.4 mM KCl, 1.8 mM CaCl_2_, 1.0 mM MgCl_2_ and 5 mM glucose. The cells were also incubated with nominal divalent cation-free ([Ca^2+^]_0_) medium with 0.5 mM EGTA, but not CaCl_2_ and MgCl_2_, a condition that induces opening of hemichannels. The cells were exposed to 50 μM EtBr for 15 minutes and EtBr uptake was measured.

For IL-1 experiments, human cortical or mouse spinal cord astrocyte cells, prepared based on our previous publication (23), were plated onto 12-well plates and incubated with DMEM/F12 medium plus 10% FBS until confluence. IL-1β (1 nM) was added to medium, and cells were incubated for 24 hours. On the day of the experiment, MHC1 (66.7 nM) or Cx43E2 antibody (6.7 nM) was added to medium, and cells were incubated for 30 minutes prior to addition of EtBr (50 μM) for 5 minutes. Cells were washed with Dulbecco’s PBS (DPBS) 3 times and fixed with 2% PFA/PBS for 10 minutes (16). For each experimental condition, at least 3 microphotographs of fluorescence fields were imaged under identical exposure and light intensity with an Olympus LUCPlan FLN 20×, work distance of 0.45 mm dry objective in an inverted microscope (Olympus IX70) with a rhodamine filter. The image was obtained with a Zeiss digital camera (AxioCamMR) using AxioVision 4.1 software (Zeiss) hooked with a homemade adapter. The image analysis was conducted off-line with NIH ImageJ. For each condition, we measured the average pixel density of the EtBr signal of 25 random cells and 2 background regions of interest. The background was the density of pixels taken from 2 areas without cells and then averaged. The final value was obtained by subtracting mean background value from cell value.

Gap junction intercellular coupling was determined at 25°C using the scrape-loading dye transfer technique in confluent mouse astrocyte primary cultures in absence or presence of 66.7 nM of MHC1 or 100 μM CBX for 3 or 24 hours at 37°C. Briefly, cells were washed twice with DPBS, and scrape-loading was performed by scraping cells with a sharp razor containing the gap junction–permeable fluorescent dye 1% Lucifer yellow (LY) and 1% of gap junction–nonpermeable fluorescent dye rhodamine dextran 10 kDa (RD). After 5 minutes, cells were washed 4 times with the DPBS and then fixed with 2% PFA for 10 minutes. Fluorescence images were captured using an inverted fluorescent microscope (BZ-X800, Keyence). Experiments were repeated 3 times and data were quantified by measuring fluorescence areas of 3 fields using NIH ImageJ software. Quantification of changes in dye coupling under different treatments was performed by measuring the fluorescence area in square millimeters of the LY fluorescence minus RD fluorescence.

### Immunolabeling of cell surface expressed Cx43.

MLO-Y4 cells, provided by Lynda F. Bonewald while at UTHSCSA, were cultured on collagen-coated glass coverslips for 48 hours and were incubated with 13.3 nM in-house rabbit polyclonal Cx43E2 antibody or 33.5 nM in-house MHC1 for 3 hours at 4°C. The cells were rinsed 3 times with cold DPBS with Ca^2+^ and Mg^2+^, fixed with 1% PFA for 15 minutes. The cells treated with Cx43E2 or MHC1 antibody were blocked with blocking solution without Triton X-100 (2% donkey serum, 2% fish skin gelatin, and 1% BSA in PBS) for 30 minutes. The primary antibodies were detected using 1:500 Alexa Fluor 488–conjugated donkey anti-rabbit antibody, Alexa Fluor 488–conjugated donkey anti-human antibody (Invitrogen, Thermo Fisher Scientific), or WGA 594 (Invitrogen, Thermo Fisher Scientific) for 1 hour. Cells on coverslips were mounted using Vectashield mounting medium (H-1000, Vector Laboratories) and sealed. Fluorescence imaging was performed using a confocal laser scanning microscope (Fluoview; Olympus Optical). For cells in suspension, MLO-Y4 cells cultured in 60 mm collagen-coated dishes were rinsed with DPBS and nonenzymatically detached and disaggregated with TrypLE (Thermo Fisher Scientific). The cells were rinsed in 2% FBS in DPBS and counted. A total of 30,000 MLO-Y4 cells in 100 μL were incubated with Fc Block (BD Biosciences catalog 553142) (1:100) for 20 minutes, incubated with 66.7 nM MHC1 for 1 hour, rinsed 3 times, and incubated with 2% FBS in DPBS plus FC blocker and 1:500 Alexa Fluor 488–conjugated mouse anti-human IgG1 Fc antibody (Invitrogen, Thermo Fisher Scientific, catalog A-10631). The cells were rinsed 3 times and fixed. The cells were centrifuged at 1000*g* for 3 minutes at 4°C and resuspended in 100 μL in PBS. Twenty microliters of sample was mounted on coverslips using Vectashield mounting medium and sealed for microscopy (BZ-X800 Keyence).

### Immunohistochemistry.

Animals were euthanized with isoflurane and perfused intracardially with cold PBS followed by 4% PFA in PBS (pH 7.4). Approximately 10 mm of spinal cord centered over the injury was dissected and fixed in 4% PFA overnight and then transferred to 30% sucrose for 24 hours. Cryo-sections of spinal cords were prepared as transverse tissue sections (from dorsal to ventral side) and sagittal sections (from head to tail). For preparation of transverse tissue sections, we kept the spinal cord vertical and measured the distance from rostral end to injury central point (ICP). We cut the sections from rostral to caudal and harvested them in 3 sites (ICP, ICP+0.65 mm, ICP+2 mm). For continuous transverse sections, we prepared a 10 μm thickness section in every 100 μm for a total 2 mm length centered on the injury site. For sagittal sections, we measured the width of each spinal cord and prepared sections in 3 sites (midline, midline+0.4 mm, midline+0.8 mm) on both left and right sides, 5 continuous sections for each site. All tissue sections had a thickness of 10 μm.

Immunohistochemistry was carried out using the following antibodies: for primary antibodies, chicken anti-GFAP (MilliporeSigma AB5541, 1:1000 dilution, or AB5804, 1:500 dilution) for detection of astrocyte activation and chicken anti-MAP2 (Abcam, ab5392, 1:1000 dilution) for detection of neurons; for secondary antibodies, goat anti-human Alexa Fluor 488 (Thermo Fisher Scientific, A-11013) and donkey anti-human Alexa Fluor 594 (Thermo Fisher Scientific, SA5-10128) to label MHC1 antibody and goat anti-chicken Alexa Fluor 568 secondary antibody (Thermo Fisher Scientific, A-11041) to detect primary antibodies against GFAP and MAP2. Immunolabeling of GFAP or MAP2 was determined using FIJI-ImageJ. Thresholds for signals in the images were set at 1.5× background gray values. A region of interest within 100 μm of the spinal cord lesion was selected for analysis. The mean intensity and total number of pixels above threshold were measured, and immunolabeling was determined as the intensity × total pixels above threshold. Results were expressed as a percentage of sham IgG–treated values. A modified EC myelin staining protocol (66) to differentiate white matter and gray matter was used to quantify the proportion of spared tissue in serial transverse sections of the injured spinal cord. Briefly, air-dried sections were cleaned and hydrated before being placed in EC solution (0.16% EC, 0.4% FeCl_3_, 0.4% aqueous H_2_SO_4_) for 12 minutes. The sections were then rinsed in water and incubated for 20 seconds in 0.5% NH_4_OH. The reaction was terminated with rinses in water before sections were dehydrated and covered with a coverslip. Spared tissue was defined and the percentage of white matter and gray matter was individually calculated by dividing the spared white or gray matter by the total area of the spinal cord on a given section using FIJI-ImageJ (31).

### Statistics.

Statistical analysis was performed using GraphPad Prism 5 statistics software. All data are presented as mean ± SEM. Unpaired parametric 1-tailed *t* test, general linear model, linear mixed model of repeated measures with antedependent covariance structure of time, linear mixed model of repeated measures with autoregressive covariance structure of time, 2-way repeated measures ANOVA with time, and 1-way ANOVA with Bonferroni’s test were used for statistical analysis. *P* < 0.05 was considered statistically significant.

### Study approval.

All described animal protocols were reviewed and approved by UTHSCSA IACUC, in accordance with policies dictated by the Office of Animal Welfare at the NIH, USA.

## Author contributions

CZ, ZY, AM, MAR, SG, ZA, NLS, and JXJ designed research studies. CZ, ZY, AM, MAR, SG, GB, DJW, VB, AG, and WX conducted the experiments. CZ, ZY, AM, MAR, SG, GB, DJW, VB, AG, WX, NZ, ZA, NLS, and JXJ acquired and analyzed data. CZ, NLS, and JXJ wrote the original manuscript, which was edited and approved by CZ, ZY, AM, MAR, SG, GB, DJW, VB, AG, WX, NZ, MJM, ZA, NLS, and JXJ. ZA, NLS, and JXJ acquired funding and supervised the study. Both CZ and ZY made equal contributions; CZ started the project earlier than ZY and so is listed first.

## Supplementary Material

Supplemental data

## Version 1 03/08/2021

Electronic publication
