# Supplementary material for: Inhibition of astrocyte hemichannel improves recovery from spinal cord injury
Source: JCI Insight. 2021 Mar 8;6(5):e134611. doi: 10.1172/jci.insight.134611 (PMC8021110; doi:10.1172/jci.insight.134611)
Supplement: Supplemental data [file jciinsight-6-134611-s021.pdf]

Figure S1: Detection of Cell Surface Cx43. (A) Live MLO-Y4 mouse osteocyte cell line were incubated with 13.3 nM Cx43E2 (top Panels) or 333.5 nM MHC1 (bottom panels) for 3 hrs at 4˚C. The cells were fixed with 1% PFA and incubated with respective secondary antibodies (1:500) conjugated with Alexa 488 (green signal) or WGA (red signal) for 1 hr at 4˚C. Bar 30 µM. (B) MLO-Y4 cells were detached and disaggregated with TryplE and keep in suspension in 2% FBS in DPBS. The cells were incubated for 1 hr at 4˚C with 66.7 nM MHC1 and then incubated with secondary antibody (1:500) Alexa 488 (green signal). 20 µl of cell suspension was mounted and imaged. MHC1 antibody binds Cx43 present on the cell surface of attached or suspended MLO-Y4 cells. Bar 100 µM.

Figure S2. MHC1 has no effect on pannexin channels. MDA-MB231 cells, which express pannexin1, but not Cx43, were treated with 250 µM ATP that induces the opening of pannexin channels. Dye uptake assay was performed by incubating with 50 µM EtBr in the absence or presence of 10 µg/ml MHC1, 100 µM CBX or 1 mM probenecid (PBC). One-way-ANOVA was used in statistical analysis. Data are presented as mean±SEM of three independent experiments, each experiment had 3-6 repeats. ***, $P < 0.001$.

Figure S3: Quantification of spinal cord astrocytes. Spinal cord astrocytes were isolated and subjected to immunocytochemistry for neural markers at passage 1. (A) Representative immunocytochemistry of astrocytes immunolabelled for intermediate filament proteins and astrocytic markers vimentin and GFAP. (B) Quantification of percentage of cells from spinal cord astrocyte preparation immunolabelled with the indicated neural markers. Results were obtained by counting the total number of labeled cells as a percentage of cells on a single coverslip. The total number of cells counted is noted on the table.

Figure S4. Hemichannel activity at SCI region. Evans Blue (EB) dye was IP injected 15 min before IP injection with 25 mg/kg MHC1 30 min after SCI with the Model 2 impact were sacrificed and perfused before isolation of spinal cords. Frozen tissue sections at transverse orientation were prepared and EB dye uptake was detected by fluorescence microscopy (upper panels). The level of dye uptake at injured region was quantified by NIH Image Software. The results are presented as mean±SEM. SCI+Saline (n=3), SCI+MHC1 (n=3). Unpaired t-test (one tailed) was used in statistical analysis. *, $P<0.05$.

Figure S5. Hemichannel activity at perilesional SCI region. Evans Blue (EB) dye was IP injected 15 min before IP injection with 25 mg/kg MHC1 after SCI with the Model 2 impact. Mice were sacrificed and perfused before isolation of spinal cords. Frozen tissue sections at transverse orientation were prepared and EB dye uptake was detected by fluorescence microscopy (upper panels). The level of dye uptake at <1.5 mm away from injured region was quantified by NIH Image Software. The results are presented as mean±SEM. SCI+Saline (n=3), SCI+MHC1 (n=3). Unpaired t-test (one tailed) was used in statistical analysis. **, $P<0.01$; ***, $P<0.001$.

Figure S6. Hemichannel activity at distal SCI region. Evans Blue (EB) dye was IP injected 15 min before IP injection with 25 mg/kg MHC1 after SCI with the Model 2 impact. Mice were sacrificed and perfused before isolation of spinal cords. Frozen tissue sections at transverse orientation were prepared and EB dye uptake was detected by fluorescence microscopy (upper panels). The level of dye uptake at >1.5 mm away from injured region was quantified by NIH Image Software. The results are presented as mean±SEM. SCI+Saline (n=3), SCI+MHC1 (n=3). Unpaired t-test (one tailed) was used in statistical analysis.

Figure S7. Gliosis reduction by MHC1 in a compression SCI model. For the compression model, Dumont SS Forceps (13.5cm length, standard tip, Fine Science Tools, Foster City CA, USA) squeezed the spinal

cord by holding the forceps perpendicular to the sagittal plane of the cord and squeezing the cord gently between the forceps tips until the forceps closed (10s) before release. This induced a complete lesion in the coronal plane of the spinal cord. Fourteen days post SCI, spinal cords were collected and the frozen sections were immunolabeled with anti-GFAP antibody (Millipore AB5541, 1:1000 dilution) for detection of astrocyte activation. Fluorescence signals were quantified by NIH Image Software. The results are presented as mean±SEM. Sham+IgG (n=5), SCI+IgG (n=4), Sham+MHC1 (n=7), SCI+MHC1 (n=3). One-way-ANOVA with Bonferroni's test was used in statistical analysis.

Figure S1

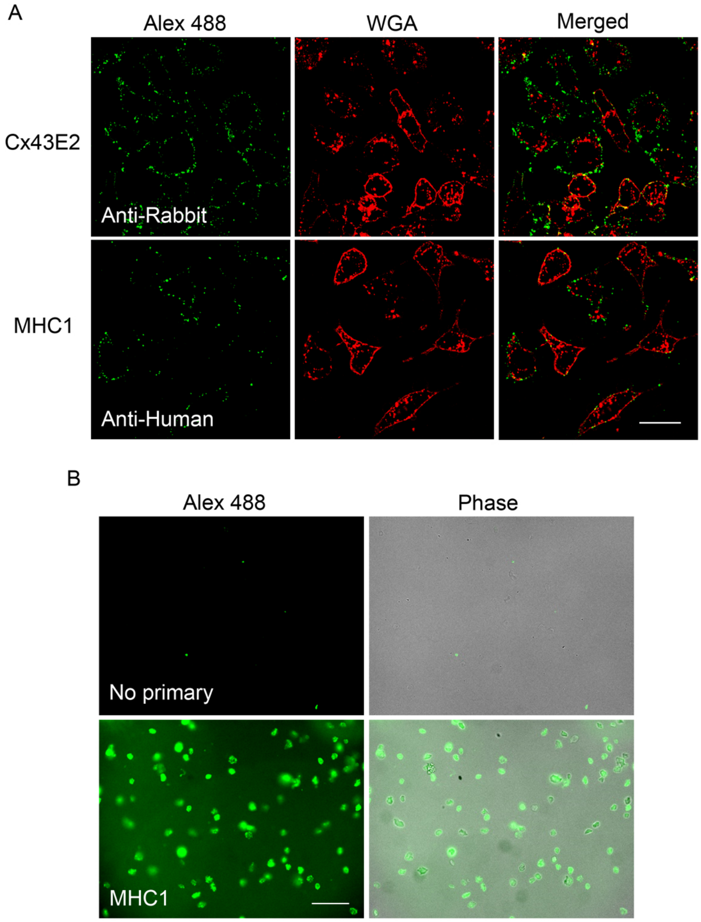
A
Alex 488
WGA
Merged
Cx43E2
Anti-Rabbit
MHC1
Anti-Human
B
Alex 488
Phase
No primary
MHC1

Figure S2

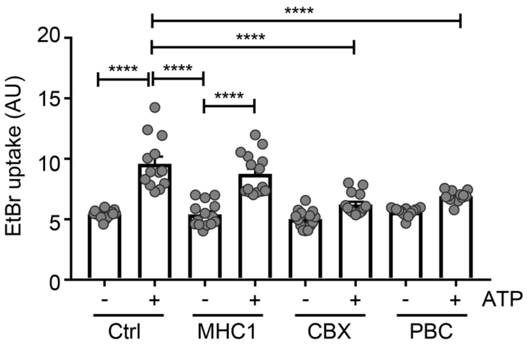

Figure S3

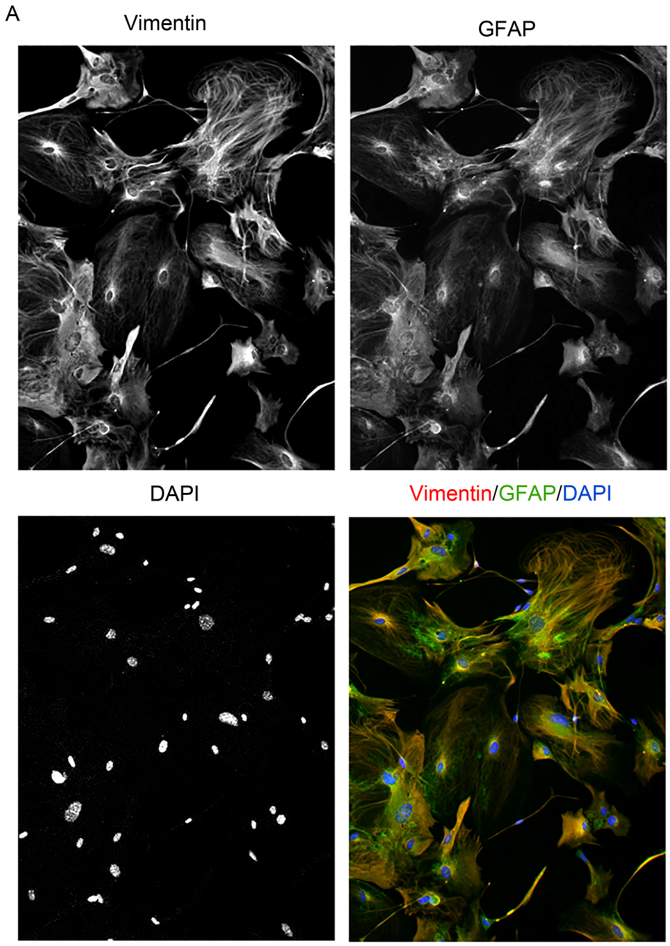

B

Cellular Marker	% labeled	
GFAP	96%	of 299
Vimentin	86.00%	of 44
S100B	100.00%	of 47
GalC	2.70%	of 36
CD11b	5.00%	of 36
MAP2	1.80%	of 57
Nestin	3.80%	of 26

Figure S4

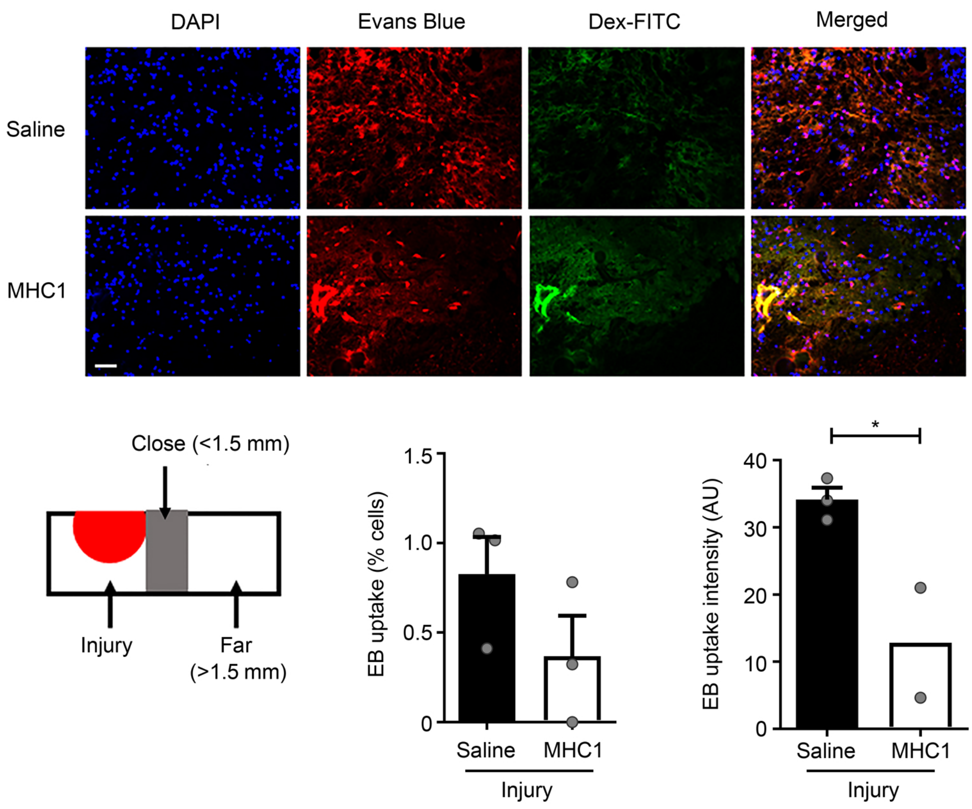
DAPI
Evans Blue
Dex-FITC
Merged
Saline
MHC1
Close (<1.5 mm)
Injury
Far
(>1.5 mm)
EB uptake (% cells)
1.5
1.0
0.5
0
Saline
MHC1
Injury
*
EB uptake intensity (AU)
40
30
20
10
0
Saline
MHC1
Injury

Figure S5

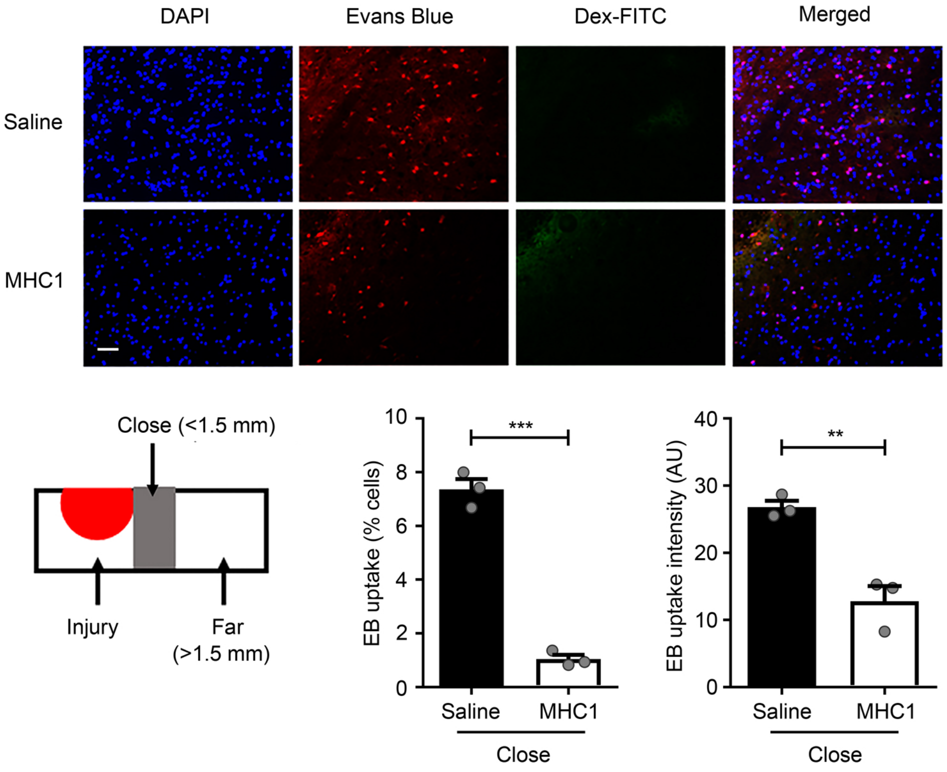
DAPI
Evans Blue
Dex-FITC
Merged
Saline
MHC1
Close (<1.5 mm)
Injury
Far
(>1.5 mm)
EB uptake (% cells)
10
8
6
4
2
0
***
Saline
MHC1
Close
EB uptake intensity (AU)
40
30
20
10
0
**
Saline
MHC1
Close

Figure S6

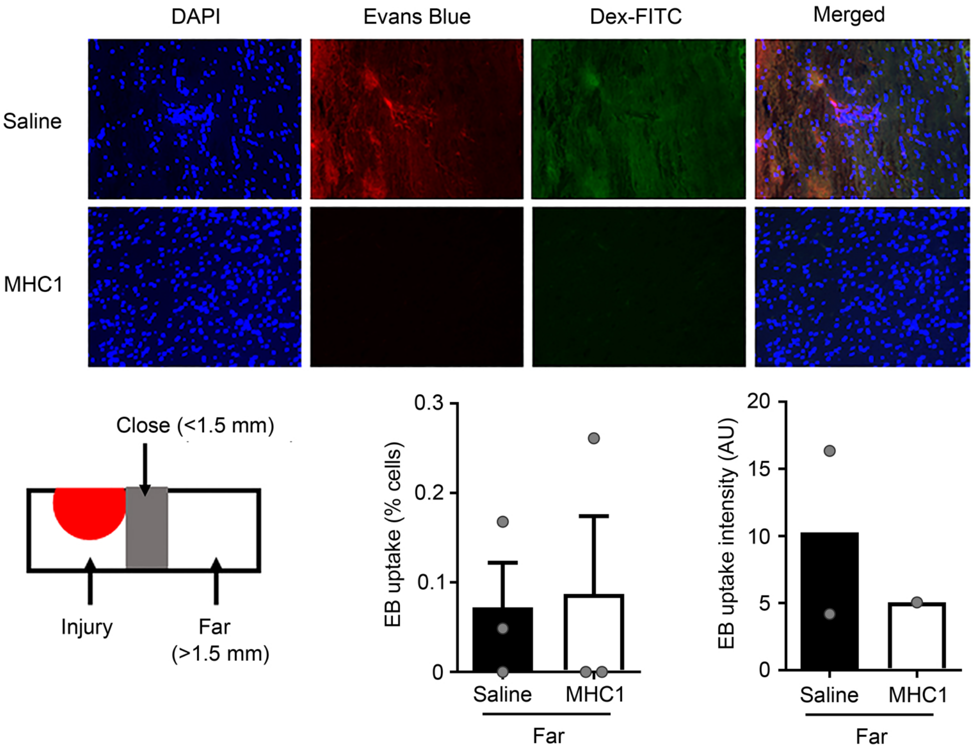
DAPI
Evans Blue
Dex-FITC
Merged
Saline
MHC1
Close (<1.5 mm)
Injury
Far
(>1.5 mm)
0.3
0.2
0.1
0
EB uptake (% cells)
Saline
MHC1
Far
20
15
10
5
0
EB uptake intensity (AU)
Saline
MHC1
Far

Figure S7

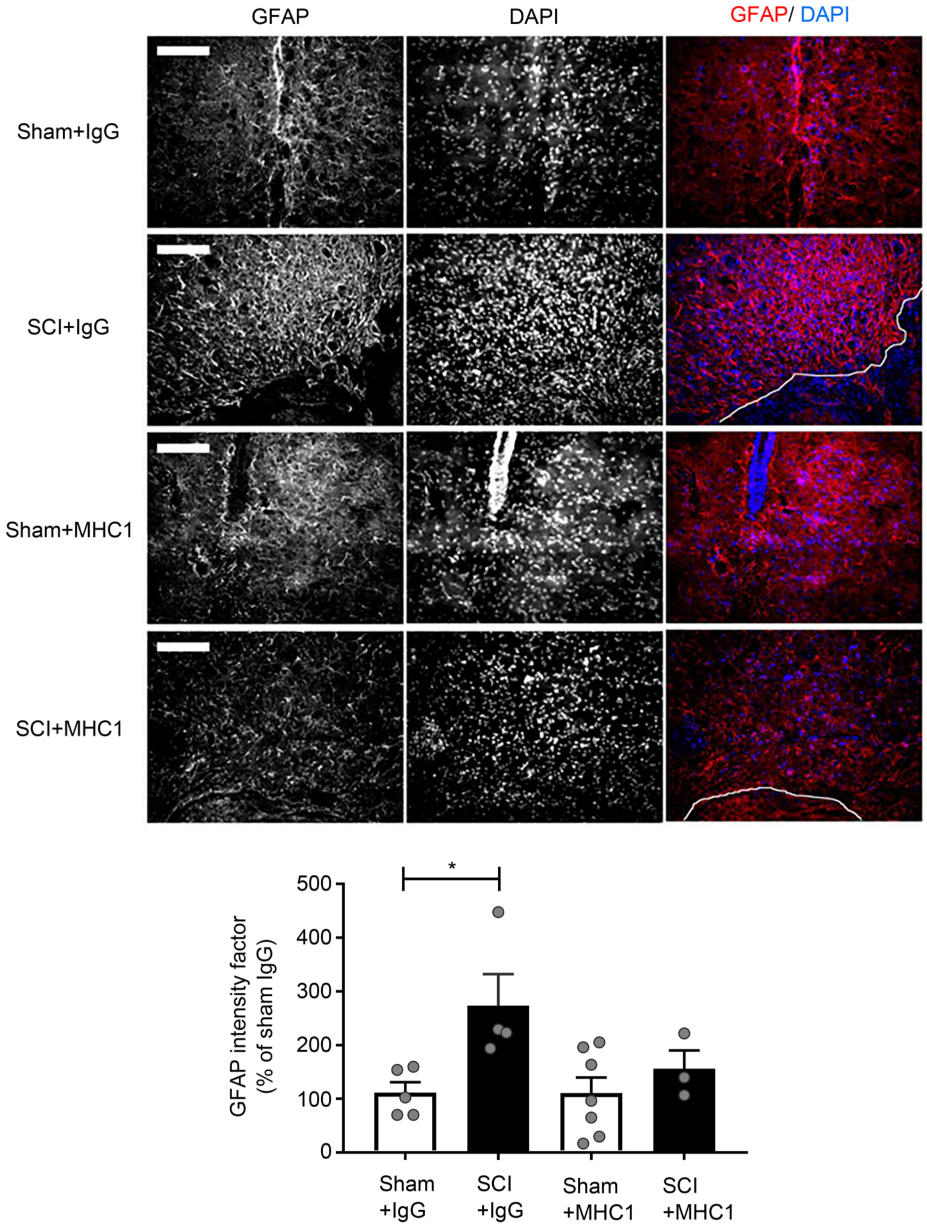

GFAP
DAPI
GFAP/ DAPI
Sham+IgG
SCI+IgG
Sham+MHC1
SCI+MHC1
*
500
400
300
200
100
0
GFAP intensity factor
(% of sham IgG)
Sham
+IgG
SCI
+IgG
Sham
+MHC1
SCI
+MHC1
